# Freezing-Tolerant Supramolecular Adhesives from Tannic Acid-Based Low-Transition-Temperature Mixtures

**DOI:** 10.1021/acsmaterialslett.4c01212

**Published:** 2024-07-17

**Authors:** Pablo A. Mercadal, Maria del Mar Montesinos, Micaela A. Macchione, Sergio D. Dalosto, Karina L. Bierbrauer, Marcelo Calderón, Agustín González, Matias L. Picchio

**Affiliations:** † Departamento de Química Orgánica, Facultad de Ciencias Químicas, Universidad Nacional de Córdoba, 5000 Córdoba, Argentina; ‡ Instituto de Investigación y Desarrollo en Ingeniería de Procesos y Química Aplicada (IPQA-CONICET), 5000 Córdoba, Argentina; § Departamento de Recursos Naturales, Facultad de Ciencias Agropecuarias, Universidad Nacional de Córdoba, 5000 Córdoba, Argentina; ∥ Centro de Investigaciones en Bioquímica Clínica e Inmunología (CIBICI-CONICET), Departamento de Bioquímica Clínica, Facultad de Ciencias Químicas, Universidad Nacional de Córdoba, 5000 Córdoba, Argentina; ⊥ Centro de Investigaciones y Transferencia de Villa María (CIT Villa María-CONICET-UNVM), X5900LQC Villa María, Córdoba, Argentina; # Instituto de Física del Litoral (IFIS-Litoral, CONICET-UNL), Güemes 3450, 3000 Santa Fe, Argentina; ∇ Centro de Excelencia en Productos y Procesos de Córdoba, Gobierno de la Provincia de Córdoba, Pabellón CEPROCOR, Santa Maria de Punilla, 5164 Córdoba, Argentina; ○ Consejo Nacional de Investigaciones Científicas y Técnicas (CCT Córdoba), 5000 Córdoba, Argentina; ◆ POLYMAT, Applied Chemistry Department, Faculty of Chemistry, University of the Basque Country UPV/EHU, Paseo Manuel de Lardizábal 3, 20018 Donostia-San Sebastián, Spain; ¶ IKERBASQUE, Basque Foundation for Science, Plaza Euskadi 5, 48009 Bilbao, Spain

## Abstract

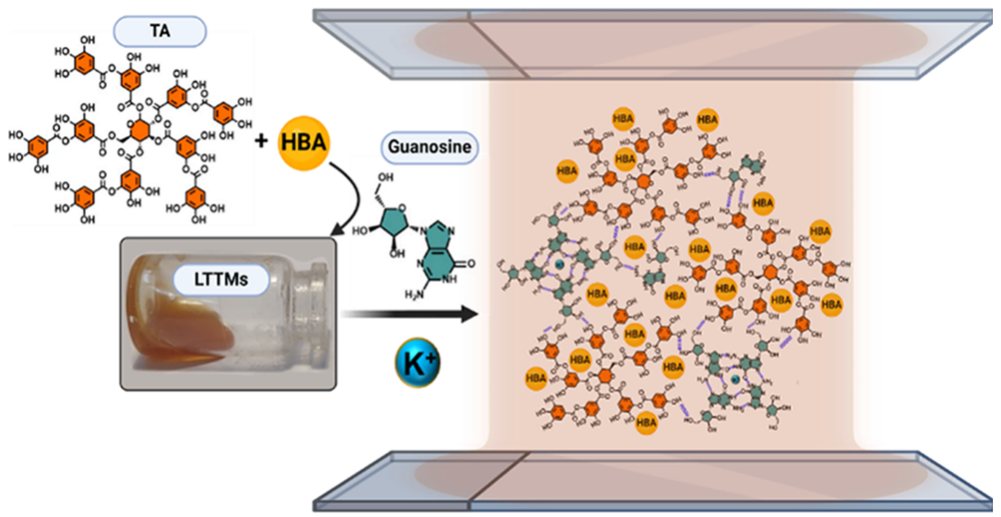

Natural polyphenols like tannic acid (TA) have recently emerged as multifunctional building blocks for designing advanced materials. Herein, we show the benefits of having TA in a dynamic liquid state using low-transition-temperature mixtures (LTTMs) for developing freezing-tolerant glues. TA was combined with betaine or choline chloride to create LTTMs, which direct the self-assembly of guanosine into supramolecular viscoelastic materials with high adhesion. Molecular dynamics simulations showed that the structural properties of the material are linked to strong hydrogen bonding in TA–betaine and TA–choline chloride mixtures. Notably, long-term and repeatable adhesion was achieved even at −196 °C due to the binding ability of TA’s catechol and gallol units and the mixtures’ glass transition temperature. Additionally, the adhesives demonstrated injectability and low toxicity against fibroblasts *in vitro*. These traits reveal the potential of these systems as bioadhesives for tissue repair, opening new avenues for creating multifunctional soft materials with bioactive properties.

Natural polyphenols have attracted significant attention over the past few years as functional building molecules in materials science due to their fascinating chemical and biological properties.^1−4^ Their chemical structure, rich in quinones, catechol, and gallol groups, among others, allows them to orchestrate a broad range of interactions. These interactions include hydrogen bonding, π–π stacking, dynamic covalent bonds, and metal–ligand coordination, which have been exploited to create functional materials.^5,6^ They are ubiquitous in the plant kingdom, and their structural motif can also be found in some marine organisms.^7−9^ For instance, catechol and gallol units in the proteins of mussels and ascidians are responsible for their extraordinary underwater adhesion.^10−13^ Furthermore, polyphenols have demonstrated valuable therapeutic properties, including antioxidant, anti-inflammatory, and antimicrobial activity.^14−17^

Considering the current central role of these biomolecules in different areas, we see huge potential for using them in low-transition-temperature mixtures (LTTMs). These solvents are mixtures of pure compounds that exhibit a glass transition temperature instead of a melting point.^18,19^ Kroon et al. first reported these unusual liquids,^20^ and some authors consider them as a subclass of deep eutectic solvents (DES) where the negative deviations from the thermodynamic ideality are so strong that the melting point of the mixture is entirely suppressed.^21,22^ Like DES, many LTTMs have high ionic conductivity, low volatility, excellent thermal stability, good biocompatibility, and biodegradability, with the system choline chloride (ChCl): lactic acid (1:2 mol ratio) being an archetypical case.^3,23^ Recently, we have discovered that combining ChCl with tannic acid (TA), maybe the most used polyphenol nowadays, results in the formation of LTTMs with metal coordination ability.^24^ TA allows adhesion to various substrates, such as organic and inorganic materials and hydrophilic and hydrophobic surfaces.^25−28^ Furthermore, we have also demonstrated that the TA: ChCl mixture displays antioxidant and antibacterial activity.^29^ However, the concept of TA-based LTTMs to fabricate soft materials remains unexplored. In this regard, an appealing application for this type of mixture could be the design of freezing-tolerant and solvent-free adhesive systems, such as tissue glues, where TA’s therapeutic features give additional valuable properties.

Indeed, the development of solvent-free supramolecular adhesives to avoid polluting solvent evaporation and eventual collapse of the material has attracted significant attention from both scientific and industry communities.^30−33^ For instance, Xie et al. reported an adhesive based on catechin and poly(ethylene glycol), resulting in a viscous supramolecular polymer.^34^ A biodegradable block copolymer–TA glue was recently explored for hair transplantation surgery.^35^ Additionally, eutectic mixtures from biopolymers and eutectogels based on low-molecular-weight gelators (LMWGs) have been proposed as supramolecular adhesive systems.^27,36,37^ In this sense, guanosine (G) is a popular nucleoside that can serve as LMWG via quadruplex formation mediated by borate ion/cation complexation.^38−40^ In this supramolecular chemistry, we believe that TA could replace the borate ion given its great affinity by −OH groups,^41^ identifying G as an excellent candidate for building dynamic adhesives with phenolic LTTMs.

Inspired by the state-of-the-art, in this letter, we present freezing tolerant and solvent-free supramolecular adhesive systems (S-F_SAS) based on innovative TA-based LTTMs and G. The LTTMs employed consist of mixtures of TA with ChCl or betaine (Bet) at 1:20 mol ratio. As displayed in Figure 1A, these LTTMs are homogeneous liquids with a brown color at room temperature. It is worth noting that this is the first report for Bet/TA LTTM fabrication. After obtaining the LTTMs, different amounts of G and potassium hydroxide (KOH) were added to the solvents. The mixture of G and K^+^ in water forms the guanosine quartet (G4) complex, consisting of four G hydrogen-bonded together.^42^ This complex or the individual G molecules might interact with TA-based solvents to form the S-F_SAS (Figure 1A). The selected G concentrations were 1, 2, 3, and 4% w/v for adhesives with ChCl/TA (*x*% G-ChCl/TA) and 0.5, 1, 2, and 3% w/v for adhesives with Bet/TA (*x*% G-Bet/TA). As displayed in Figure 1B, the inversion test revealed a nonflowable behavior for the selected concentrations. In contrast, TA-based LTTMs, where G molecules are not included, show flowable behavior. The flowability test results for other concentrations are presented in Figure S1A in the Supporting Information (SI). The lowest G concentration to form a nonflowable material was found to be 0.5% for G-ChCl/TA and 0.25% for G-Bet/TA samples (Figure S1B, left). The upper concentration limit for the complete dissolution of G molecules was 5% for G-ChCl/TA and 4% for G-Bet/TA, respectively (Figure S1B, right). We observed that the inversion test showed a flowable behavior if K^+^ is not added to the S-F_SAS samples (Figure S1C). These results suggest that the G4 tetramer functions as a thickener in the material, which is consistent with previously reported works.^42,43^ It is worth noting that the presence of single G molecules that do not form the G4 complex is not ruled out. As displayed in Figure 1C (left), FT-IR spectra of both LTTMs show a centered broad band attributed to the O–H ν around 3286 cm^–1^ for ChCl/TA and 3230 cm^–1^ for Bet/TA. In the spectra of the S-F_SAS samples, these bands are shifted to higher wavenumbers (see Table S1), indicating H-bonding interactions between the LTTMs and G. This observation aligns with previous reports on the cross-linking capability of TA.^29,44−46^ Notably, in the absence of KOH, the broad absorption band attributed to the O–H stretching vibration remains at the same position as in the LTTMs (Figure S2), further confirming the importance of H-bonding in these systems. The band at around 1720 cm^–1^ is present in both LTTMs and S–F_SAF and corresponds to the carbonyl stretching vibration of TA. We also identified a band at 1610–1630 cm^–1^ belonging to the C–C skeletal vibrations of TA in both LTTMs, while the Bet/TA spectrum (Figure 1C, right) exhibits a shoulder located at 1589 cm^–1^ attributed to the contribution of both aromatic C–C skeletal vibrations of TA and COO^–^ of Bet.^47^ In S-F_SAS based on Bet/TA, the maximum of these bands is shifted to lower wavenumbers and broadened as the percentage of G increases in the S-F_SAS, which may also indicate the contribution of H-bonding interactions between the solvent and G. SEM images reveal that the surface morphology of 4% G-ChCl/TA and 3% G-Bet/TA samples correspond to nonporous and smooth dense materials (Figure 1D). These observations also indicated that the formation of intermolecular hydrogen bonds plays a role in the macroscopic structure of supramolecular adhesive materials.^34,36^ Interestingly, the absence of entangled fibrils suggests that in these systems, the G4 complexes are not stacked with each other, forming the so-called G-quadruplexes.^39,42,43,48^

**Figure 1:**
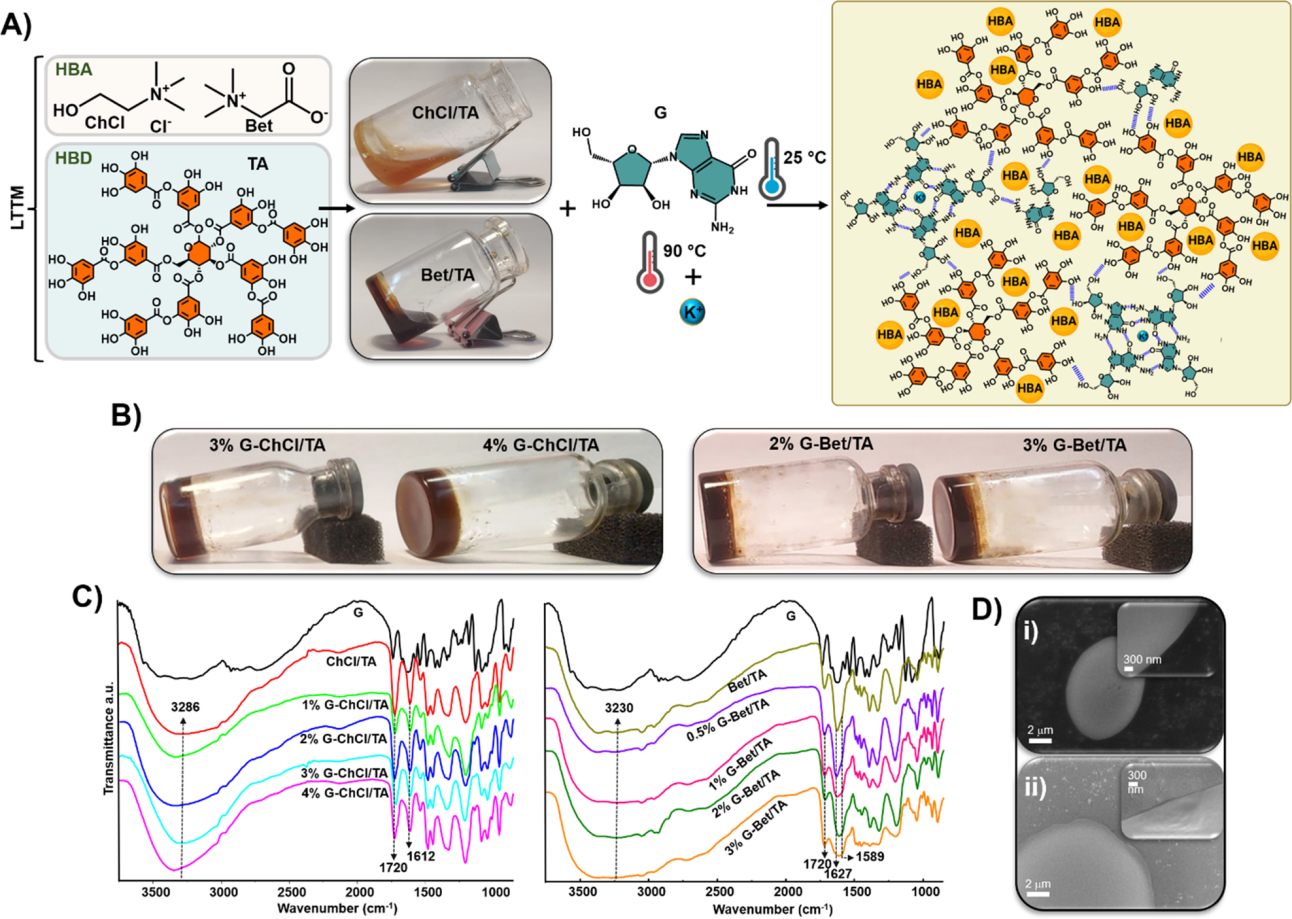
A) Scheme of the preparation of LTTMs and the proposed interactions between TA and G. HBA: hydrogen bond acceptor; HBD: hydrogen bond donor. B) Photographs of as-prepared G-ChCl/TA (left) and G-Bet/TA (right) supramolecular adhesive systems. C) FT-IR spectra of G powder, pure LTTMs and the as-prepared G-ChCl/TA (left) or G-Bet/TA (right) supramolecular adhesive systems. D) SEM images of a micro drop of 4% G-ChCl/TA (i) and 3% G-Bet/TA (ii) supramolecular adhesives.

We used molecular dynamics (MD) and first-principles to uncover the various interactions among TA, G, and ChCl or Bet. These methods allow us to understand the atomistic extent of the hydrogen bonds, Van Der Waals forces, and electrostatic interactions. We first analyze the interactions between the various components of the S-F_SAS based on Bet/TA and G, which, as mentioned before, stabilize the structures and define the adhesion and viscoelastic properties. Hydrogen bonding can occur between the −COO^–^ group of Bet and the −OH groups of the gallol and catechol moieties of TA, as well as with the −OH groups of G. Figure 2A displays the structure of one TA molecule extracted from the MD simulation of S-F_SAS based on Bet/TA. The nine carbonyl moieties of a TA molecule are buried, and the access of Bet or G molecules to a close distance (less than 4 Å) is shielded by the OH groups of the phenolic moieties (Figures 2B,C). As a result, their IR frequencies around 1720 cm^–1^ are minimally affected by Bet molecules, as seen in Figure 1C. The OH moieties of TA can rotate easily, facilitating the interaction with the −COO^–^ group of Bet to form a stable interaction. Curiously, the phenolic groups adopt a more open position than when water molecules surround them.^49^ Presumably, this structural conformation partially contributes to the elastic response of the S-F_SAS with Bet. At the molar ratio TA: Bet of 1:20, the TA molecules interact with each other, forming a kind of poly(TA) arrangement with Bet molecules in between, functioning as electrostatic cross-linkers. Figures S3–S5 show in detail the typical interactions among these constituents of S-F_SAS. The Bet molecules form a shell around the TA molecules with a strong hydrogen bond interaction Bet-TA, where the Bet molecules act as hydrogen bond acceptors for the −OH groups of the TA molecule. Besides, the electrostatic interaction between Bet molecules is weak due to the shielding effect produced by its methyl group but sufficient to induce a nearly uniform cover around the TA molecules, see Figure S6. A detailed study of the interactions between TA and G molecules and Bet and G molecules is presented in the SI. Surprisingly, MD simulations also revealed that G4 may only be found in a low amount in the S-F_SAS, as G molecules could hardly form a tetramer structure due to their low concentration and hindered mobility in the LTTMs. This result could explain why G-quadruplexes are not formed, as evidenced by SEM analysis as discussed above. However, those few G4 formed were almost structurally stable during the simulation, primarily due to the interactions with TA, which partially prevented interactions with Bet and acted as an anchor, as shown in Figure 2D. Additionally, the intramolecular interaction is strong, resembling a quasi-covalent bond. Note that if G4 are even present at low concentrations, they play a key role in structuring the S-F_SAS, as nonflowable materials were only obtained with the addition of K^+^ ions, which facilitate and stabilize the self-assembly of G monomers into G4 structures.^50^ Moreover, the presence of amino groups in G and K^+^ can interact with the gallic moieties of TA through cation-π interactions helping to stabilize the structure and preventing the flowability of the material, in line with the findings by Cui et al.^51^

**Figure 2:**
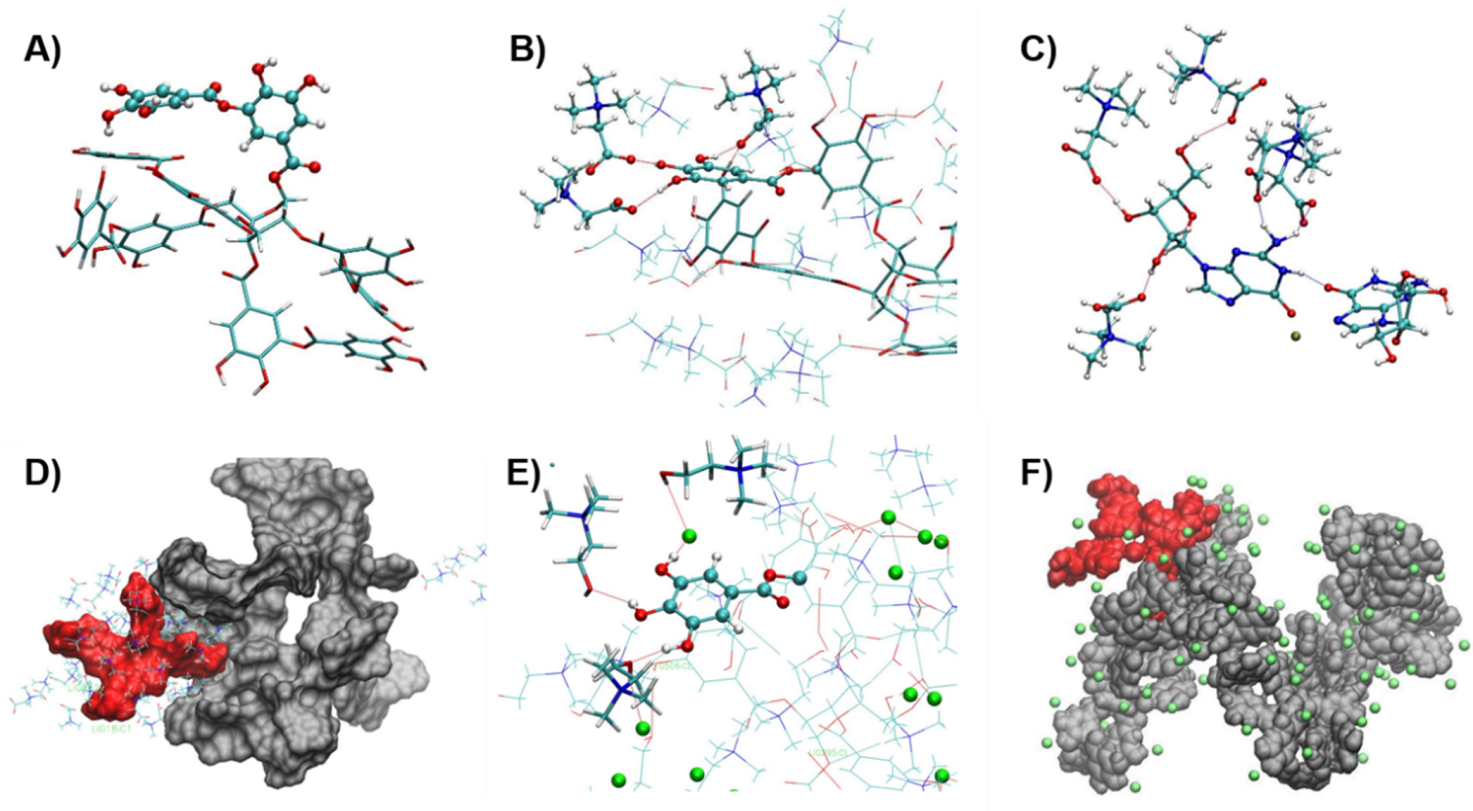
A) Representative snapshot showing TA molecule. B) Bet molecules interacting with TA. Here, two phenolic groups are shown and the rest of TA molecule and Bet molecules were deleted to help visualization. C) One G molecule interacting with Bet molecules. D) The G4 is presented in red color and surrounded by a shell of Bet (stick representation, 3 Å to the Bet surface) and TA molecules (surface representation in gray color. E) Detail showing one gallic moiety of the TA molecule interacting with a Cl ion (green color) and the choline cation. The rest of the molecules were removed. F) TA molecules (surface representation in color gray) and G molecules (surface representation in color red). The Cl ions (color green) closer than 4 Å to the surface of TA and G molecules are shown. The Ch cations were removed for clarity.

Similar to the case of S-F_SAS with Bet molecules, the nine carbonyl groups of TA molecules are buried and exhibit weak interactions with both the chloride anion and the choline cation in the ChCl/TA mixtures. Consequently, their IR frequencies are minimally affected by the presence of ChCl, which is in line with the FTIR results. The electronegative chloride anion shields the TA molecule from the choline cation, resulting in a surrounding shell of molecules that is less dense than what we observe with Bet molecules. These anions are located close to the 25 −OH groups of each TA molecule (Figure 2E). Additionally, the diffusion of these chloride ions is hindered by their interaction with the hydroxyl groups of the TA molecules. We found that the number of Cl anions surrounded by a TA molecule is 18 ± 2. The role of Cl^–^ ions is 2-fold: they disrupt the intramolecular TA-TA, TA-Ch^+^ cation, TA-G, and G-G interactions, thereby producing a shielding effect and reducing the density of the mixture, which is presumed to be responsible for the elastic response of the TA-G-ChCl system (Figure 2F). A detailed study of the interactions between TA and G molecules and ChCl and G molecules is presented in the SI (Figures S7–S10).

The thermal properties of the samples were first examined via thermogravimetric analysis (TGA), where it was observed an increase in the decomposition temperature of G after their incorporation into both LTTMs, which is another evidence of the affinity between G and the nonvolatile solvents (see Figures S11A–D and Table S2). The decomposition temperature of the samples until 5% (T_5%_) was taken as the upper limit for the DSC studies. As displayed in Figure 3A, ChCl/TA LTTM exhibits thermal stability without glass transition temperature (T_g_) in the range of studied temperatures (−40 to 120 °C), which agrees with our previous reports.^24,29^ In the case of the 4% G-ChCl/TA, a T_g_ is observed at 34.2 °C, indicating that the G molecule interacts with the LTTM and induces a change between the glassy and liquid microscopic state.^52^ Bet/TA LTTM shows a T_g_ at 20.8 °C, which shifts to 23.2 °C for the 3% G-Bet/TA sample. Figure S11E,F shows the remaining DSC scans, while Table S2 presents their respective T_g_ values, where a subtle increase was observed as the G amount increased. The flexible and viscoelastic properties of the prepared S-F_SAS allow their injectability, which is valuable for biomedical applications (Figure 3B). Then, we explored the viscoelastic properties of the S-F_SAS by small-amplitude oscillatory shear. Interestingly, the elastic modulus (*G*′) of the systems is lower than the viscous modulus (*G*″) in the whole range of tested amplitude and frequency, demonstrating that the S-F_SAS have a viscoelastic liquid nature (Figures 3C,D and S12A–D). A gel state was possibly not reached in these systems due to the limited formation of G4 structures, attributed to the restricted diffusion of G in the LTTMs, thereby hindering their assembly.

**Figure 3:**
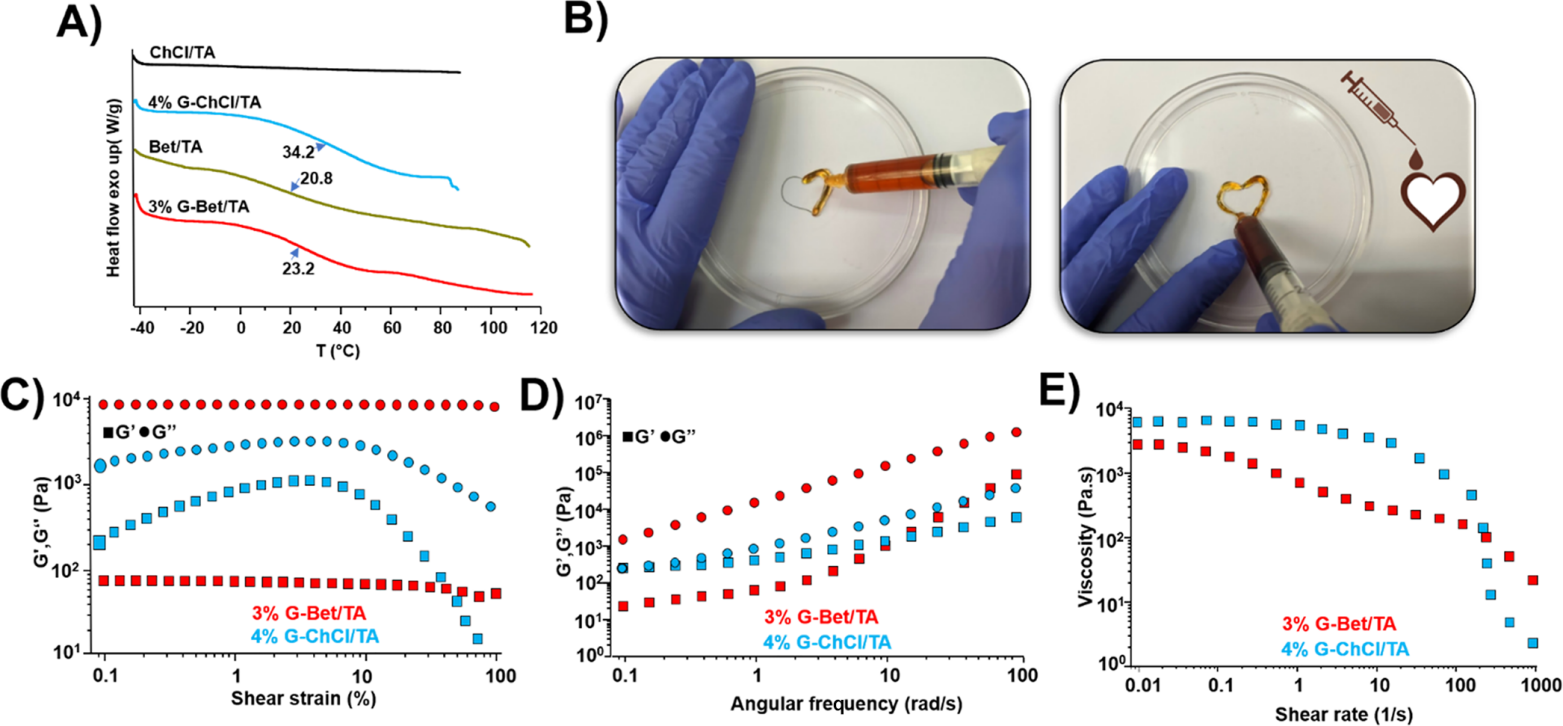
A) DSC scans of NADES and S-F_SAS with the respective values of T_g_ (blue arrows). B) Photography of the injectable properties of 4% G-ChCl/TA (left) and 3% G-Bet/TA (right) sample. C) Strain sweep from 0.1 to 100% at a frequency of 1.0 rad s^–1^. D) Frequency sweep analysis from 0.1 to 100 rad/s at a constant strain of 0.1%. E) Viscosity as a function of the shear rate for 4% G-ChCl/TA and 3% G-Bet/TA sample.

It is worth noting that the *G*′ and *G*″ moduli of S-F_SAS based on ChCl/TA show a sharp decrease in strain amplitude, whereas these moduli are almost constant for Bet/TA, indicating that this S-F_SAS is a more robust and stable material. This behavior could be explained by the fact that in the TA/Bet-based S-F_SAS, electrostatic cross-linking is accomplished through poly(TA)/Bet arrangement, while in TA/ChCl-based materials, this effect is hampered due to that Cl^–^ ions disrupt the intramolecular TA-TA interactions, as revealed by MD results. The viscosity as a function of the shear rate shows that the S-F_SAS have a non-Newtonian fluid behavior. S-F_SAS based on ChCl/TA experiences a sudden decrease in the viscosity at high shear rates, while S-F_SAS with Bet/TA shows a gradual decrease until 110 1/s, after which it decreases abruptly (Figures 3E and S12E,F). This shear-thinning behavior confirms the injectability properties of the S-F_SAS. The first inspection of the adhesive properties of the prepared S-F_SAS was performed by determining the force needed to detach these viscoelastic materials from a steel surface. As displayed in Figure 4A,D, the adhesive stress of S-F_SAS based on Bet/TA LTTM is higher than that for ChCl/TA mixture. These differences in the adhesiveness are attributed to the zwitterionic Bet molecule that presents the same positively charged ammonium moiety as ChCl and a carboxylate group that improves the adhesion through noncovalent interactions.^53,54^ Indeed, the adhesive stress value of pure Bet/TA LTTM is slightly higher than pure ChCl/TA (Figure S13). However, the magnitude of these values is negligible compared to that obtained after adding G, confirming that this nucleoside is fundamental in structuring the materials and improving the adhesion properties. In the same S-F_SAS series, as the concentration of G increases, the tackiness values increase until a maximum for 3% G-ChCl/TA or 2% G-Bet/TA samples. Subsequently, the tackiness decreases, likely due to a fewer available polar groups caused by the intermolecular interactions of TA and G (violet and brown columns of Figure 4B,E, respectively). Importantly, as the concentration of G increases, the adhesion energy values of S-F_SAS based on ChCl/TA or Bet/TA also increase until they reach 3% and 2%, respectively. This effect is more significant for the S-F_SAS based on Bet/TA. After reaching these values, the adhesion energy remains almost the same (pink and purple columns in Figure 4B,E). As shown in Figure 4C, the samples 3% G-ChCl/TA and 4% G-ChCl/TA exhibit the highest compression stress values of the series and a stress vs strain curve with two distinct segments: an initial rigid part and a second part with reduced stiffness that indicate the collapse of their structure. On the other hand, the remaining S-F_SAS samples tend to increase in stiffness as the strain increases. In the case of S-F_SAS based on Bet/TA, the samples with the highest compression stress values are 1% G-Bet/TA and 2% G-Bet/TA (Figure 4F), significantly outperforming the TA/ChCl-based S-F_SAS. Once again, poly(TA)/Bet electrostatic cross-linking in the TA/Bet-based systems seems to greatly impact the macroscopic properties of the adhesives.

**Figure 4:**
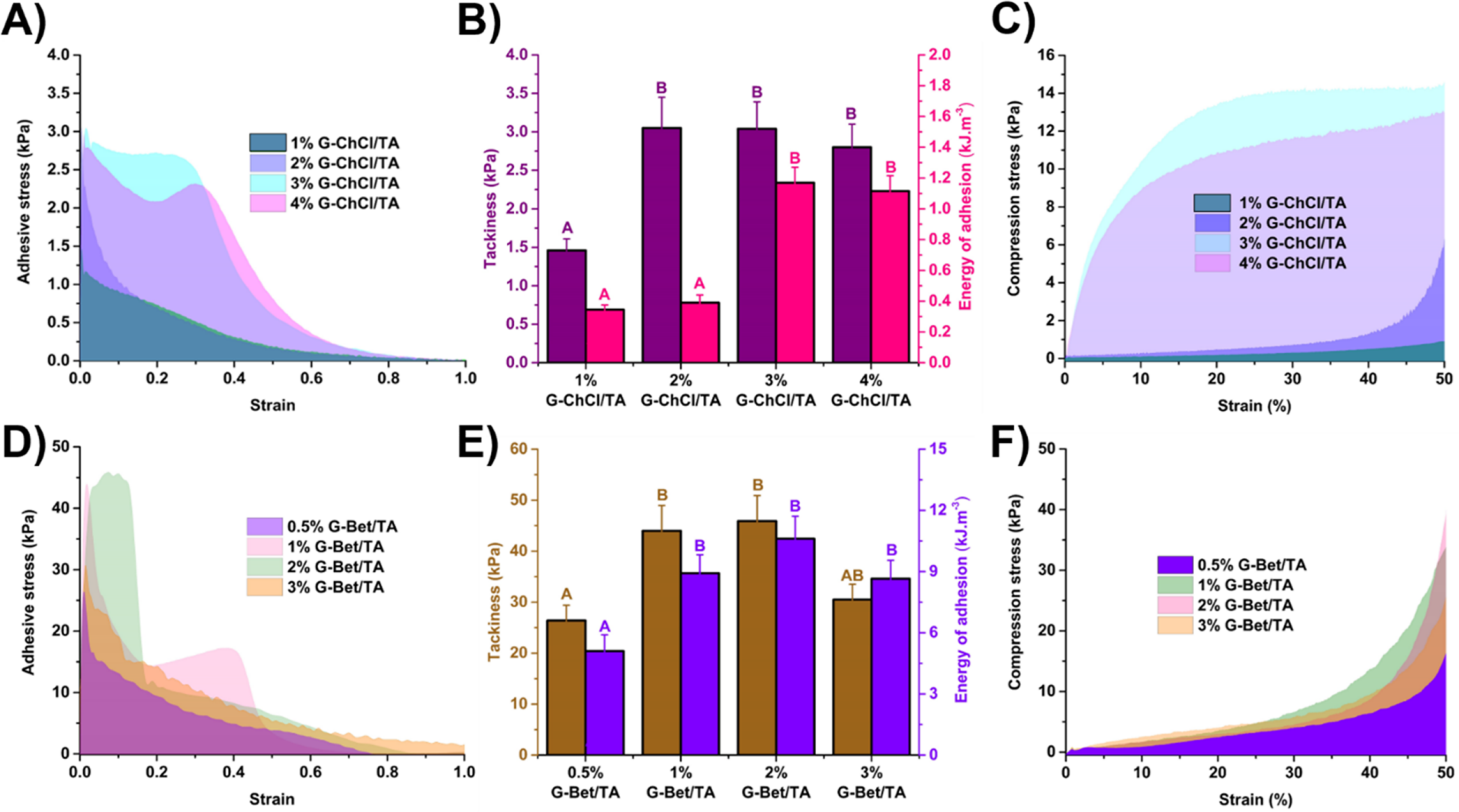
Adhesive stress vs strain curves of the S-F_SAS based on ChCl/TA (A) and Bet/TA (D) LTTMs. Tackiness and Energy of adhesion values of the S-F_SAS based on ChCl/TA (B) and Bet/TA (E) NADES. Stress vs strain compression curves of the S-F_SAS based on ChCl/TA (C) and Bet/TA (F) NADES. Two bar values with the same letter are not significantly different (*p* ≥ 0.05) according to Tukey’s test (*n* = 5).

Based on the results of the above adhesion stress vs strain curves, we chose the 2% G-Bet/TA and 3% G-ChCl/TA S-F_SAS to study the adhesive behavior on different substrates. As displayed in Figure 5A, 2% G-Bet/TA S-F_SAS can adhere to two identical substrates (e.g., wood/wood), similarly to 3% G-ChCl/TA S-F_SAS (Figure S14). Furthermore, the adhesion of S-F_SAS to two different surfaces, e.g., nitrile/wood, can be observed in Figure 5B for 3% G-ChCl/TA and Figure S15 for 2% G-Bet/TA. The adhesion stress of S-F_SAS was measured as the maximum force required to pull apart the identical surfaces divided by the contact area.

**Figure 5:**
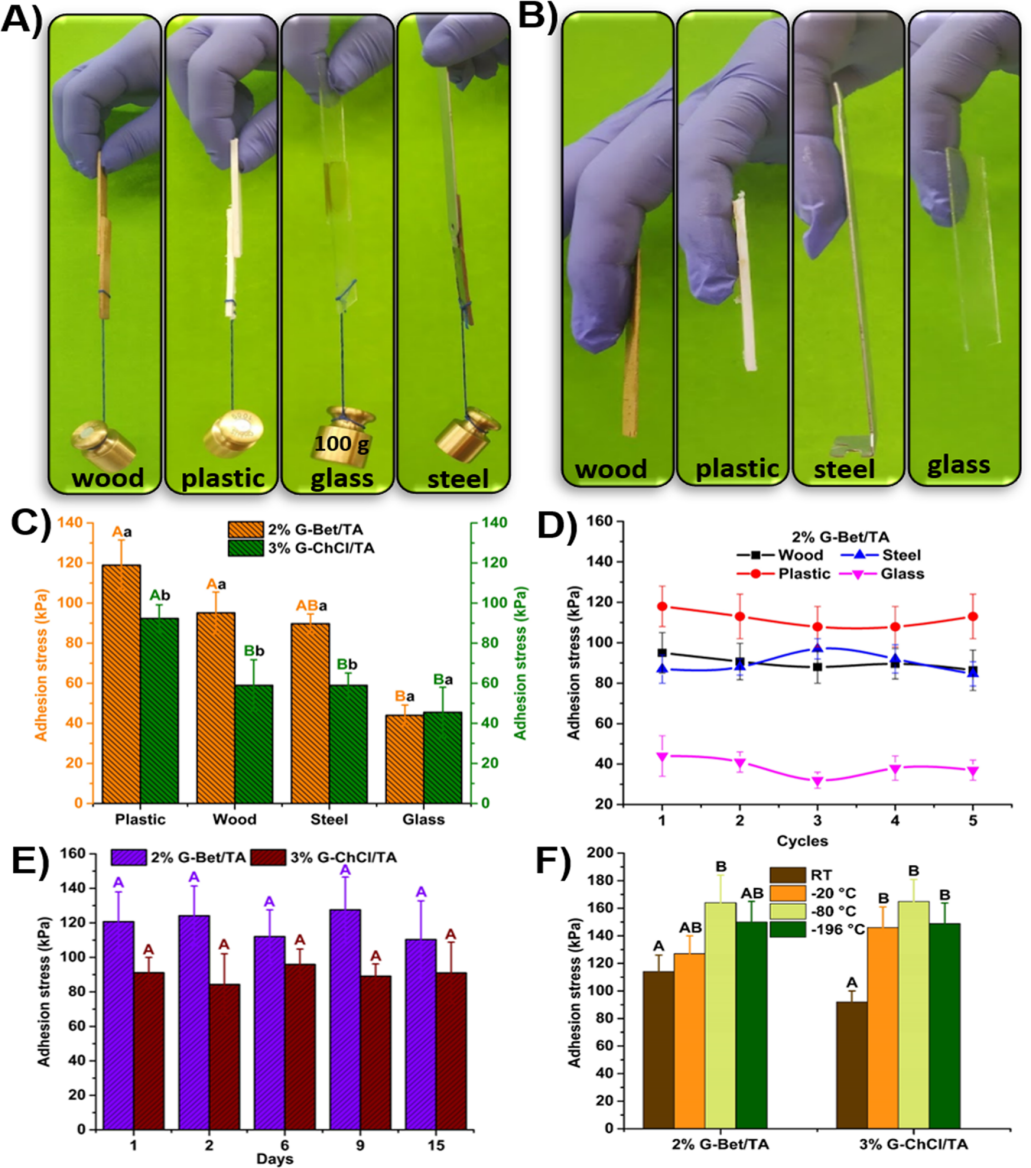
A) Adhesion behaviors of the 2% G-Bet/TA adhesive for attaching various substrates. B) Adhesion behaviors of the 3% G-ChCl/TA adhesive for attaching nitrile to various substrates, including wood, plastic, glass, steel. C) Adhesion stress of the 2% G-Bet/TA and 3% ChCl-Bet/TA adhesives on different substrates at 25 °C. D) Cycling tests of the 2% G-Bet/TA adhesive on different substrates at 25 °C. E) Adhesion stress of the 2% G-Bet/TA and 3% ChCl-Bet/TA adhesives at different time periods. F) Adhesion stress of the 2% G-Bet/TA and 3% ChCl-Bet/TA adhesives at 25, −20, −80, and −196 °C on plastic substrates. Two bar values with the same letter are not significantly different (*p* ≥ 0.05) according to Tukey’s test (*n* = 5). Major letters are statistical significances between different substrates for the same S-F_SAS whereas minor letters are for the same substrate but different S-F_SAS.

As displayed in Figure 5C, 2% G-Bet/TA S-F_SAS exhibits, after 24 h of forming the joint between the two surfaces, higher values of adhesion stress than 3% G-ChCl/TA for plastic, wood, and steel substrates, probably due to its higher cohesiveness by TA-TA intramolecular cross-linking and extra noncovalent interactions provided by the Bet molecule.^53,54^ For the glass surface, both S-F_SAS show similar values of adhesion stress since this surface is less rough; thus, it is difficult to form strong adhesive interactions.^55^ Furthermore, the adhesion strength on pigskin at 50% of RH was 91.6 ± 9 kPa and 59.6 ± 6 kPa for 2% G-Bet/TA and 3% G-ChCl/TA, respectively. Notably, these values are comparable to those observed on steel and wood surfaces and surpass those on glass. Both S-F_SAS exhibit good adhesion on pigskin at higher relative humidity levels (82% RH) without statistical difference from the experiment at 50% RH (Figure S16), demonstrating the remarkable tolerance of these materials to aqueous environments. The cyclic adhesion test for the substrates using 2% G-Bet/TA S-F_SAS is shown in Figure 5D. The results show that this S-F_SAS can keep similar adhesion stress values with slight adhesion loss until the fifth cycle of adhesive attaching/detaching on plastic, steel, wood, and glass surfaces minor to 10%. However, for the case of using 3% G-ChCl/TA S-F_SAS, the adhesive shows losses of adhesion until the fifth cycle of 16, 30, 77, and 56% for plastic, steel, wood, and glass surfaces, respectively (Figure S17). The reduced adhesion behavior can be attributed to the lower cohesion of these S-F_SAS compared to the Bet/TA-based S-F_SAS and the presence of Bet molecules in the last one. Figure 5E displays the temporal evolution of adhesion stress values for both S-F_SAS at room temperature and relative humidity. The results reveal that both adhesives consistently uphold their adhesive properties over the entire 15-day period. An essential feature of an adhesive that expands its field of applications is the ability to operate at temperatures different from room conditions and avoid solvent evaporation. No macroscopic changes were observed after lyophilizing the 2% G-Bet/TA and 3% G-ChCl/TA S-F_SAS for 30 h (Figure S18). In addition, the weight loss values were (0.50 ± 0.05)% and (0.42 ± 0.10)% for the 2% G-Bet/TA and 3% G-ChCl/TA S-F_SAS, respectively. These negligible weight loss values indicate the antidrying nature of the S-F_SAS and their ability to avoid solvent evaporation, which is given by the low vapor pressure feature of these LTTMs. Another key property of these neoteric solvents is their antifreezing traits, expanding the applicability of these innovative S-F_SAS to harsh environments. The comparison of the adhesion stress values of the S-F_SAS at RT, −20, −80, and −196 °C on a plastic substrate is shown in Figure 5F. The results indicate an increase in the adhesion stress after the freezing process down to −80 °C, maintaining a similar value at −196 °C for both S-F_SAS samples. This increase in adhesive strength values at low temperatures is probably due to the favoring of multiple hydrogen bonding interactions provided by TA.^46,56^ Consistent with our data, Qin et al. demonstrated superior adhesive strengths at subzero temperatures in gelatin organohydrogels, which was attributed to the antifreezing and thermoplastic properties of these materials resulting from hydrogen bonding between the protein and glycerol.^57^

Considering the great potential of the S-F_SAS in tissue adhesives and other biomedical applications, we evaluated their toxicity *in vitro* cell cultures. Specifically, MRC-5 human fibroblasts were exposed to increasing concentrations of 3% G-ChCl/TA and 2% G-Bet/TA extracts for 24 h. The cytotoxicity of the S-F_SAS was assessed by determining the cell viability by flow cytometry. Cell viability of those exposed only to the culture medium was set at 100%, serving as a baseline to compare with the responses of the S-F_SAS extracts. As shown in Figure S19A, the cell viability of 3% G-ChCl/TA is higher than 2% G-Bet/TA (Figure S19B). The calculated median cytotoxic concentration (CC50) of 3% G-ChCl/TA is 25.41%, whereas it is 15.12% for 2% G-Bet/TA (Figure S19C,D). The higher cytotoxicity of 2% G-Bet/TA compared to 3% G-ChCl/TA is attributed to the different cytocompatibility of the HBA, considering that the HBD is present in the same proportion in both LTTMs and the G concentration is higher in 3% G-ChCl. These results are consistent with the findings by Jurko et al.,^58^ who demonstrated that Bet is more cytotoxic than ChCl. We also performed red blood cell tests to evaluate the potential for skin irritation of the S-F_SAS. Triton (TX-100) was used as a control, a nonionic and biodegradable surfactant less cytotoxic and irritant than cocoamidopropyl betaine, a typical emulsifier in cosmetic formulations.^59^ Figures S19E and F show the hemolysis values of the S-F_SAS normalized by the hemolytic activity of TX-100 (positive values stand for higher hemolytic activity than the control). In line with the cell viability study, the 2% G-Bet/TA sample shows a higher hemolysis percentage than 3% G-ChCl/TA, with an activity similar to TX-100 up to 5%. Although further studies are required in *ex-vivo* and *in vivo* models, these results suggest the low skin irritation potential of the S-F_SAS.

Altogether, we have identified a new type of LTTM based on the natural multifunctional polyphenol TA, serving as an innovative precursor for designing freezing-tolerant and antidrying supramolecular adhesives when combined with G nucleoside complexes. These exciting materials behave like viscoelastic liquids with *T*_g_ values around RT, showing injectability, shear-thinning properties, and excellent adhesion to various substrates. Besides, the nonvolatile nature of the LTTMs enables strong substrate interactions at RT without mass loss after several bonding/debonding cycles. Remarkably, these systems can function well even at −196 °C, maintaining their adhesive properties over extended periods. Cellular viability tests over fibroblasts demonstrated that Bet-based LTTMs exhibit higher toxicity than ChCl-based mixtures at high dosages. Given the numerous therapeutic properties of TA, we envision these supramolecular adhesives as having a promising future in the biomedical sector, and our results pave the way for exploring new bioactive LTTMs.

## Supporting Information Available

Materials and methods; Photographs of the rest of S-F_SAS; Photographs of the lower and upper limits for the formation of a solid material; Photographs of the samples without the addition of KOH; FTIR spectra of the samples without the addition of KOH; Computational study of the interactions among the constituents; TGA, Derivate Weigh (%/°C), and DSC of samples; Glass and Decomposition temperatures; Frequency, Amplitude, and Viscosity measurements; Dynamic Simulations; Adhesive and Compression stress of pure NADES; Adhesion behavior of the 3% G-ChCl/TA adhesive for attaching two same substrates; Adhesion behavior of the 2% G-Bet/TA adhesive for attaching two different substrates; Cyclic adhesion test of 3% G-ChCl/TA; Photography of samples after and before 24 h of lyophilization; Citotoxicity and hemolysis tests (PDF)

## Abbreviations

HBD: hydrogen bond donor
HBA: hydrogen bond acceptor
NADES: natural deep eutectic solvents
Bet: betaine
ChCl: Choline chloride
TA: tannic acid
G: guanosine
S–F_SAS: solvent-free supramolecular adhesive system
1% G-ChCl/TA, 2% G-ChCl/TA, 3% G-ChCl/TA and 4% G-ChCl/TA: Choline Chloride/Tannic acid NADES with different %w/v of guanosine. 0.5% G-Bet/TA, 1% G-Bet/TA
2% G-Bet/TA and 3% G-Bet/TA: Betaine/Tannic acid NADES with different %w/v of guanosine
SEM: scanning electron microscopy
DSC: differential scanning temperature
TGA: thermogravimetric analysis
T_5%_: degradation temperature at 5%
T_50%_: degradation temperature at 50%
*T*_max%_: maximum degradation temperature
Tg: glass transition temperature
*G*′: elastic modulus
*G*″: viscous modulus.
