# Supplementary material for: Freezing-Tolerant Supramolecular Adhesives from Tannic Acid-Based Low-Transition-Temperature Mixtures
Source: ACS Mater Lett. 2024 Jul 17;6(8):3726–35. doi: 10.1021/acsmaterialslett.4c01212 (PMC11307168; doi:10.1021/acsmaterialslett.4c01212)
Supplement: Supplementary file 1 — tz4c01212_si_001.pdf [file tz4c01212_si_001.pdf]

SUPPORTING INFORMATION

Freezing-Tolerant Supramolecular Adhesives from Tannic Acid-Based Low-Transition-Temperature Mixtures (LTTMs)

Pablo A. Mercadal,[1,2,3*] Maria del Mar Montesinos,[4] Micaela A. Macchione,[1,2,5] Sergio D. Dalosto[6], Karina L. Bierbrauer,[7,8,] Marcelo Calderón,[9,10] Agustín Gonzalez,[1,2*] and Matias L. Picchio[9*]

[1] Universidad Nacional de Córdoba, Facultad de Ciencias Químicas, Departamento de Química Orgánica, Córdoba 5000, Argentina.

[2] Instituto de Investigación y Desarrollo en Ingeniería de Procesos y Química Aplicada (IPQA-CONICET), Córdoba 5000, Argentina. *Email:* pablo.mercadal@unc.edu.ar; agustingonzalez@unc.edu.ar

[3] Universidad Nacional de Córdoba, Facultad de Ciencias Agropecuarias, Departamento de Recursos Naturales, Córdoba 5000, Argentina.

[4] (Universidad Nacional de Córdoba), Departamento de Bioquímica Clínica, Facultad de Ciencias Químicas, Centro de Investigaciones en Bioquímica Clínica e Inmunología (CIBICI-CONICET), Córdoba 5000, Argentina.

[5] Centro de Investigaciones y Transferencia de Villa María (CIT Villa María-CONICET-UNVM), Villa María, Córdoba X5900LQC, Argentina.

[6] Instituto de Física del Litoral (IFIS-Litoral, CONICET-UNL), Güemes 3450, Santa Fe 3000, Argentina

[7] Centro de Excelencia en Productos y Procesos de Córdoba, Gobierno de la Provincia de Córdoba, Pabellón CEPROCOR, Santa Maria de Punilla, Córdoba 5164, Argentina

[8] Consejo Nacional de Investigaciones Científicas y Técnicas, CCT Córdoba, Córdoba 5000, Argentina.

[9] POLYMAT, Applied Chemistry Department, Faculty of Chemistry, University of the Basque Country UPV/EHU, Paseo Manuel de Lardizábal, 3, Donostia-San Sebastián 20018, Spain. *Email:* matiasluis.picchiop@ehu.eus

[10] IKERBASQUE, Basque Foundation for Science, Plaza Euskadi 5, Bilbao 48009, Spain

Experimental

Materials

All chemicals were used without any further purification. Tannic acid (Bio Pack, ≥99%); Choline chloride (Sigma Aldrich, ≥99%); Betaine (Sigma Aldrich, ≥99%); Guanosine (Sigma Aldrich, ≥98%); KOH pellets (Bio Pack, ≥97%); Mili-Q water was used throughout the work.

Characterizations

LTTMs preparation

Low-transition-temperature mixtures (LTTMs) were prepared by the heating method, the most commonly used in the literature, based on mixing the HBD and HBA and heating them at 95 °C under constant stirring until a homogeneous liquid is formed. ChCl/TA LTTM was prepared using a ChCl: TA molar ratio of 20:1. Bet/TA LTTM was prepared using a Bet: TA molar ratio of 20:1, plus adding 10% water to facilitate the mixing of the components. This last mixture was heated for 3 h in an open system to evaporate the water.

Synthesis of Solvent-free supramolecular adhesives

To prepare adhesives, 3 mL of ChCl/TA LTTM and G (1, 2, 3, or 4 w/v%, based on the LTTM) were mixed at 90 °C under vigorous stirring. After the dissolution of G, solid KOH was added to the mixture, keeping a molar ratio of 2:1 G/K^+ in each case, respectively. After the dissolution of KOH, the samples were cooled at room temperature and used for further studies. The adhesive systems with Bet/TA were prepared under the same condition but utilizing a concentration of G of 0.5, 1, 2, or 3 w/v% based on the LTTM.

FTIR analysis

The samples were characterized using a Nicolet 5-SXC spectrometer coupled to a Nicolet iN10 microscope (Thermo Scientific, USA). The FTIR spectra were recorded in reflection mode by depositing the sample on a gold mirror and collecting an average of 32 scans with 4 cm^{-1} resolution, with air as the background.

Morphological characterization

The surface morphology of the adhesives was studied by scanning electron microscopy (SEM). A drop of 10 μL of the sample was attached to a double-sided carbon adhesive tape mounted on SEM stubs, coated with gold under a vacuum, and examined with an SEM microscope (Carl Zeiss - Sigma, Germany). SEM images were acquired at a magnification of 5000X and 32000X, an aperture size of 20 μm, electron high tension (EHT) of 10 kV, and a working distance of 2.7 or 2.1 mm.

Thermal properties

The thermogravimetric analysis (TGA) was performed in a Hi-Res Modulated 2950 Thermogravimetric Analyzer (TA Instruments, USA) at 10 °C/min from 25 °C to 450 °C.

Differential scanning calorimetry (DSC) analyses of the solvent-free supramolecular adhesives, LTTMs, and G were performed on a 2920 Modulated DSC (TA Instruments, USA). 5 mg of each sample was sealed in a hermetic aluminum pan and heated, with a rate of 10 °C/min, from -40 to the decomposition temperature of 5% obtained from TGA analysis for each sample. A nitrogen flow (50 mL/min) was maintained during the entire test.

Rheological tests

Rheological tests were performed using an Anton Paar rotational rheometer (Physica MCR 302, Austria). A parallel plate geometry of 25 mm in diameter was used for the different tests. Amplitude sweeps were carried out to determine the linear viscoelastic range (LVR) of samples at 25°C with a frequency of 1 Hz and strain amplitude from 0.1 to 100%. For frequency sweeps, a fixed strain was selected within the linearity range with a frequency interval of 0.1 to 100 Hz at 25°C. Samples were evaluated in duplicate.

Adhesiveness test

Adhesive stress measurements were performed using an Instron Universal Testing Instrument (model EMIC 23-5S, Norwood, MA, USA) equipped with a 5 N load cell. An open glass vessel with 2mL of the adhesive was placed with an epoxy adhesive on a fixed metal plate of the instrument. Then, the moving steel cell of 1 cm in diameter was moved slowly towards the inside of the vessel containing the sample, and a pressure of 0.8 N/cm^2 was applied for 1 min. The adhesive strength was calculated from the force recorded while detaching the adhesives from the metallic surface, using a crosshead speed of 0.05 $mm \cdot s^{-1}$. The tackiness and energy of adhesion of the samples were calculated from the maximum force needed to pull apart the sample and the area under the stress−strain curve during the probe-removing stage, respectively. The reported values correspond to the average of three measurements of the same sample.

Compression test

Compression tests were performed at a constant speed of 18 $mm.min^{-1}$ and up to a maximum deformation of 50% on cylindrical samples of 15 $\times$ 10 mm in diameter and height, respectively. All tests were performed in quintuplicate on an Instron Universal Testing Instrument (model EMIC 23-5S, Norwood, MA, USA) equipped with a 50 N load cell.

Peak bonding strength on different substrates

A total of 20 mL of 3% G-ChCl/TA and 2% G-Bet/TA samples were evenly spread on two identical substrates, each measuring 1 cm by 1 cm. The substrates included steel, plastic, glass, and wood. The two surfaces were then joined together to create a joint area. The samples were compressed at 25 kPa for 30 min and stored at room temperature for 24 h. For the adhesion stress assay on pigskin, a skin layer from the ear with a thickness of 0.5 cm was cut using a manual dermatome from Padgett Instruments. It was then scrubbed and rinsed with phosphate-buffered serum. The pigskin was stored at -20°C and thawed before use. The previously treated pieces of skin measuring 1 $\times$ 1 cm were fixed to wood substrates of identical size. Then, 20 mL of the S-F_SAS were spread on pigskin's substrates, joined together, and compressed at 25 kPa for 30 min. Finally, a batch of samples were stored at room temperature for 24 h (~50 %RH, monitored with a hygrometer, Ferplast Argentina), and another batch in a humidity chamber at 25 °C with a stable value of 82% RH for one day. Immediately after removing the samples from the humidity chamber, the measurements were taken.

The adhered substrates were separated using an Instron Universal Testing Instrument (model EMIC 23-5S, Norwood, MA, USA) equipped with a 5 N load cell. The adhesion stress was measured by dividing the maximum force required to separate the identical surfaces by the contact area. For the cyclic adhesion test, the samples were repeatedly joined and detached. In the adhesion stress assay at -20, -80, and -196 °C, the preparation of the plastic substrate was the same as mentioned above but stored at the respective temperatures in freezers (-20 and -80 °C) or liquid nitrogen (-196 °C) for 12 h. The measurements were carried out immediately after the samples were removed from storage. All assays were performed with a minimum of five replicates, and the results were averaged.

Anti-drying tests

The anti-drying property of the S-F_SAS was tested by placing them in a freeze-drying equipment RIFCOR LT-8(-50 °C,0.022 mmHg) for 30 h. The weight loss of the different samples was calculated according to the following expression

$$\% weightloss = \qquad (1)$$

where W_i and W_f are the weights of the samples before and after 30 h of lyophilization.

Computational Methods

Quantum calculations were conducted using the Gaussian 09 software package [Gaussian 09, Revision A.02, M. J. Frisch, et al., aussian, Inc., Wallingford CT, 2016.]. Geometry optimizations were performed with the hybrid WB97XD exchange-correlation functional which includes empirical dispersion[1] and the standard 6-31G(d,p) basis set.

ESP charges (charges derived from fitting a classical Coulomb model to quantum mechanical molecular electrostatic potentials) were used for molecular dynamics studies. The classical molecular dynamics simulations were carried out at a temperature of 300 K and a pressure of 1 atm using an NAMD program.[2] After a brief minimization, equilibration and production runs were performed with a time step of 1 fs. The CHARMM 22 all-atom potential function 27 was utilized, and parameters were generated using the web server http://www.swissparam.ch. The parameters are available upon reasonable request. A cutoff of 12 Å was applied for non-bonded calculations, and all bonds involving hydrogen were kept rigid using the SHAKE algorithm. The simulations included 2 ns of equilibration followed by 8 ns of production.

The initial structure of the TA molecule was obtained from the literature,[3] which corresponds to a minimum structure found in the presence of water molecules. The quality of the above parameters was partially validated using that structure with 100 Bet or ChCl molecules present. To model the LTTM systems, we chose seven TA molecules randomly distributed, followed by the addition of 190 molecules of ChCl o Bet in a cell of size 65x65x50 Å. Four guanosine molecules and a potassium atom were added, forming either a tetramer arrangement or alone within the unit cell. These systems allow us to model the stability of the tetramer arrangement and, in the second case, model the possibility of the formation of the tetramer.

We performed molecular dynamics simulations of one TA molecule in a box of ChCl and also in a box of Bet molecules to understand the solvation of TA molecules. This allows us to quantify the number of ChCl or Bet molecules interacting with TA. Besides, it enables us to identify the groups or atoms interacting with the TA molecule.

In vitro cytotoxicity study

The cytotoxicity of 3% G-ChCl/TA and 2% G-Bet/TA extracts on MRC-5 human fibroblast cells was analyzed using live–dead cell staining. MRC-5 cells from American Type Culture Collection (ATCC CCL-171) were cultured in Dulbecco's modified Eagle's medium (DMEM, Gibco, USA) supplemented with 10% fetal bovine serum (FBS, Gibco), 100 units/mL penicillin, and 100 μg/mL streptomycin, in a humidified atmosphere of 5% CO_2/95% air at 37 °C. The S-F_SAS samples were sterilized under UV light for 15 min, and then 20 mg were incubated into 10 mL of DMEM-10% FBS at 37 °C for 24 h. MRC-5 cells seeded on 24-well plates at 80% confluence were exposed to varying concentrations of eutectogels extracts using DMEM-10% FBS as a diluent. Cells exposed only to the culture medium (DMEM-10% FBS) were used as controls. After 24 h, the culture supernatant was collected, and the cells were washed with phosphate-buffered saline (PBS: 137 mM NaCl, 2.7 mM KCl, 10 mM Na_2HPO_4 and 1.8 mM KH_2PO_4, pH 7.4). The cells were then detached with 0.05% trypsin and collected by centrifugation. Cell viability was evaluated by flow cytometry according to the operating procedures of the LIVE/DEAD™ Fixable Dead Cell Stain Kit (Far-red fluorescence reactive dye, ThermoFisher Scientific, USA). Briefly, the cells were incubated with the diluted stain for 30 min at 4 °C in the dark and washed with PBS-2% FBS. Cells were acquired on BD FACSCanto II and analyzed using the FlowJo software.

Hemolysis test

Blood was collected from healthy donors into tubes containing sodium citrate (3.2%). The erythrocyte suspension was washed three times with PBS and then diluted 1:10 in

PBS to obtain a 10% erythrocyte suspension. Aliquots of 250 μL of the erythrocyte suspension were pipetted into tubes containing a range of different concentrations of S-F_SAS extracts, with a final volume of 500 μL. To prepare 3% G-ChCl/TA and 2% G-Bet/TA extracts, 20 mg of S-F_SAS was incubated in 10 mL of PBS at 37 °C for 24 h, similar to the cytotoxicity assessment. Samples were prepared in triplicate, incubated at 37 °C for 30 min, and centrifuged at 5000 g for 5 min. The percentage of hemolysis was quantified by comparing the absorbance at 540 nm of the supernatant of each sample with that of the positive control, which was completely hemolysed with 10% Triton X-100. The erythrocyte suspension exposed to PBS alone served as the negative control. The values obtained from samples treated with the S-F_SAS extracts ($OD_{S\text{-}F\_SAS}$) were normalized relative to positive (100% lysis; OD_{Triton}) and negative (OD_{PBS}) control samples to determine the percentage of hemolysis (H) using the following equation: $H (\%) = [(OD_{S\text{-}F\_SAS} - OD_{PBS}) / (OD_{Triton} - OD_{PBS})] \times 100\%$.[4]

Statistical analysis

Data for each test were statistically analyzed. The analysis of variance (ANOVA) was used to evaluate the significance of the difference between means. Turkey test was used for comparing mean values; differences between means were considered significant when $P \leq 0.05$.

Supporting Information Results

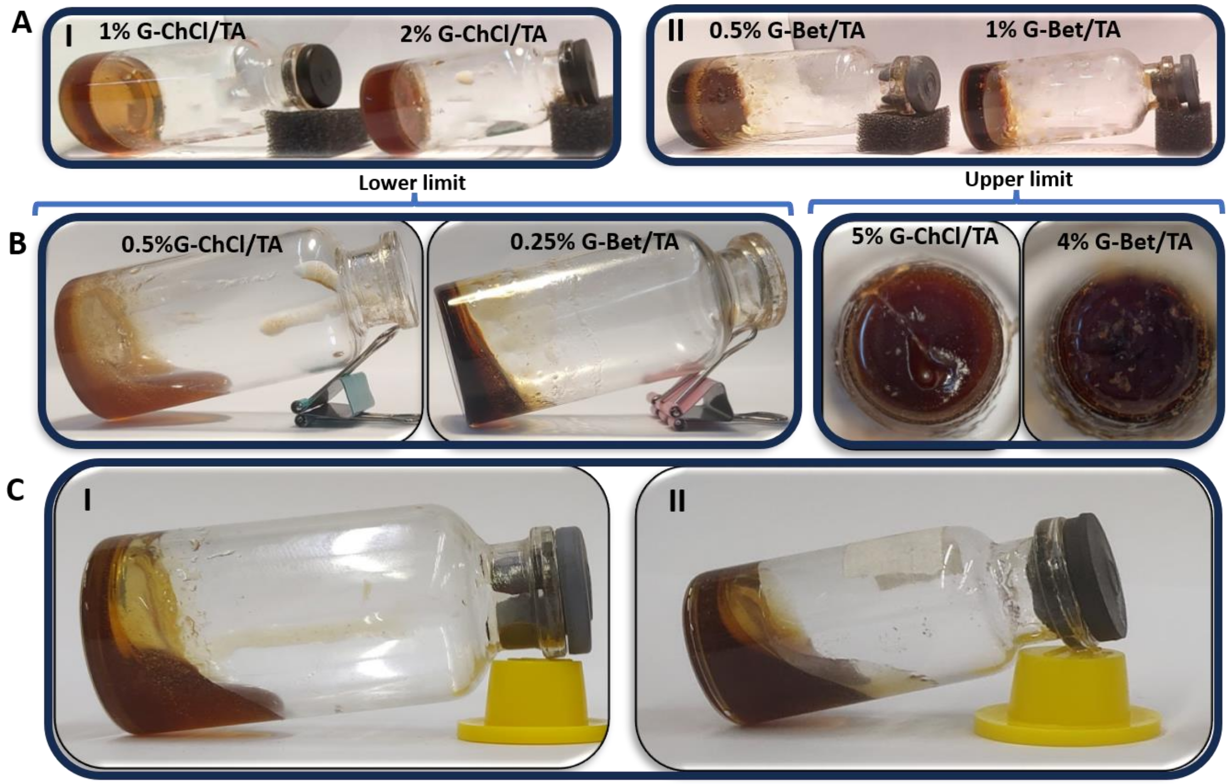

Figure S1. A) Photographs of the samples for G-ChCl (I) and G-Bet/TA (II) systems. B) Photographs of the lower and upper limit G concentration for forming a solid material. C) Photographs of the 4% G-ChCl/TA (I) and 3% G-Bet/TA (II) samples without the addition of KOH.

Table S1. FTIR bands for bending vibrations of O−H of LTTMs and S-F_SAS

Sample	band (cm^{-1})
**ChCl/TA**	**3286**
1% G-ChCl/TA	3328
2% G-ChCl/TA	3344
3% G-ChCl/TA	3295
4% G-ChCl/TA	3348
**Bet/TA**	**3230**
0.5% G-Bet/TA	3236
1% G-Bet/TA	3232
2% G-Bet/TA	3233
3% G-Bet/TA	3240

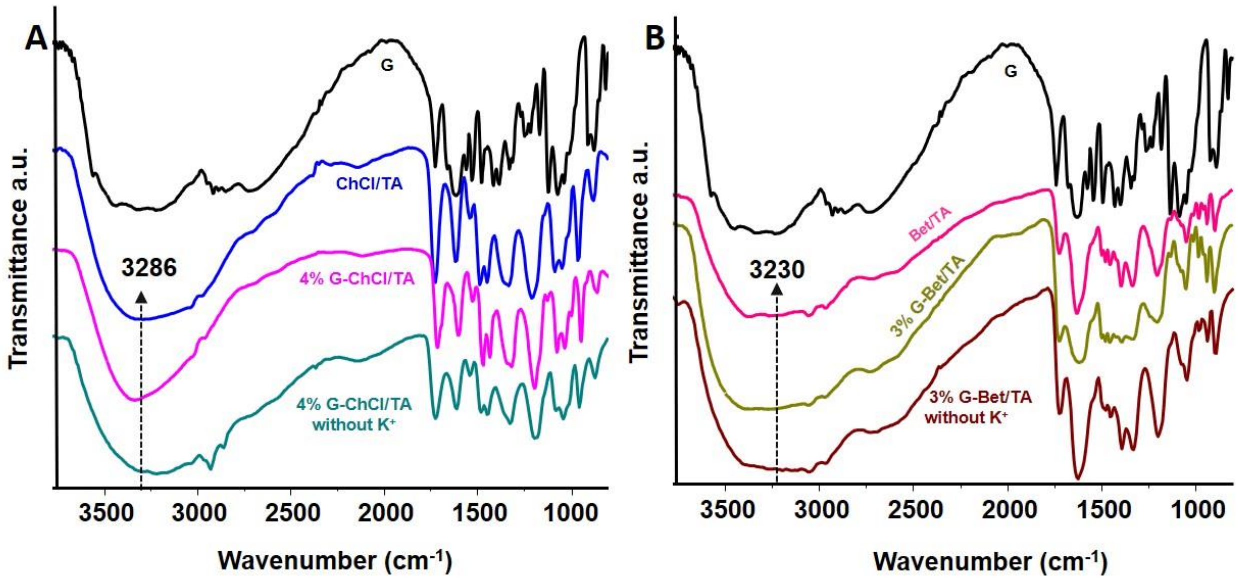

Figure S2. A) FT-IR spectra of G powder, pure ChCl/TA LTTM, and the as-prepared 4% G-ChCl/TA with and without K^+. B) FT-IR spectra of G powder, pure Bet/TA LTTM, and the as-prepared 3% G-Bet/TA with and without K^+.

Computational study of the interactions among the constituents of LTTMs.

TA and ChCl or Bet interactions involve hydrogen bonds, van der Waals (vdW) forces, and electrostatic interactions. An outstanding challenge in LTTMs is quantifying the intra- and intermolecular interactions responsible for their unique properties.

We first analyze the interactions between the various components of the LTTMs, which, as mentioned before, stabilize the structures and define the adhesion and viscoelastic properties.

TA – Bet - G

Hydrogen bonding can occur between the -COO group of Bet and the -OH groups of the phenolic moieties of TA, as well as with the -OH groups of G.

1- The nine carbonyl moieties of a TA molecule are buried, and the access of Bet molecules to a close distance (less than 4 Å) is shielded by the OH groups of the phenolic moieties. As a result, their IR frequencies are minimally affected by Bet molecules, as shown in Figure S2 and Figure 1C.

2- The OH moieties of TA can rotate easily, facilitating the interaction with the COO group of Bet to form a stable interaction. The phenolic groups adopt a more open position compared to when water molecules surround them. This structural conformation contributes in part to the elastic response of the LTTM. At the molar ratio of 1:20 (TA: Bet), the TA molecules interact with each other, forming a poly(TA) arrangement with Bet molecules in between, with Bet molecules functioning as electrostatic crosslinkers. Figure S3 shows in detail the typical interactions among the constituents of LTTMs.

3- The structural stability of the G4 was studied with two different initial positions. (i) with the tetramer arranged to include one K^+ atom surrounded by Bet and TA molecules, and (ii) starting with the G molecules separated from each other. In situation (i), we found that the tetramer remains almost structurally stable during the simulation time, primarily due to the interaction with TA molecules, which partially prevent interaction with Bet molecules and act as an anchor.

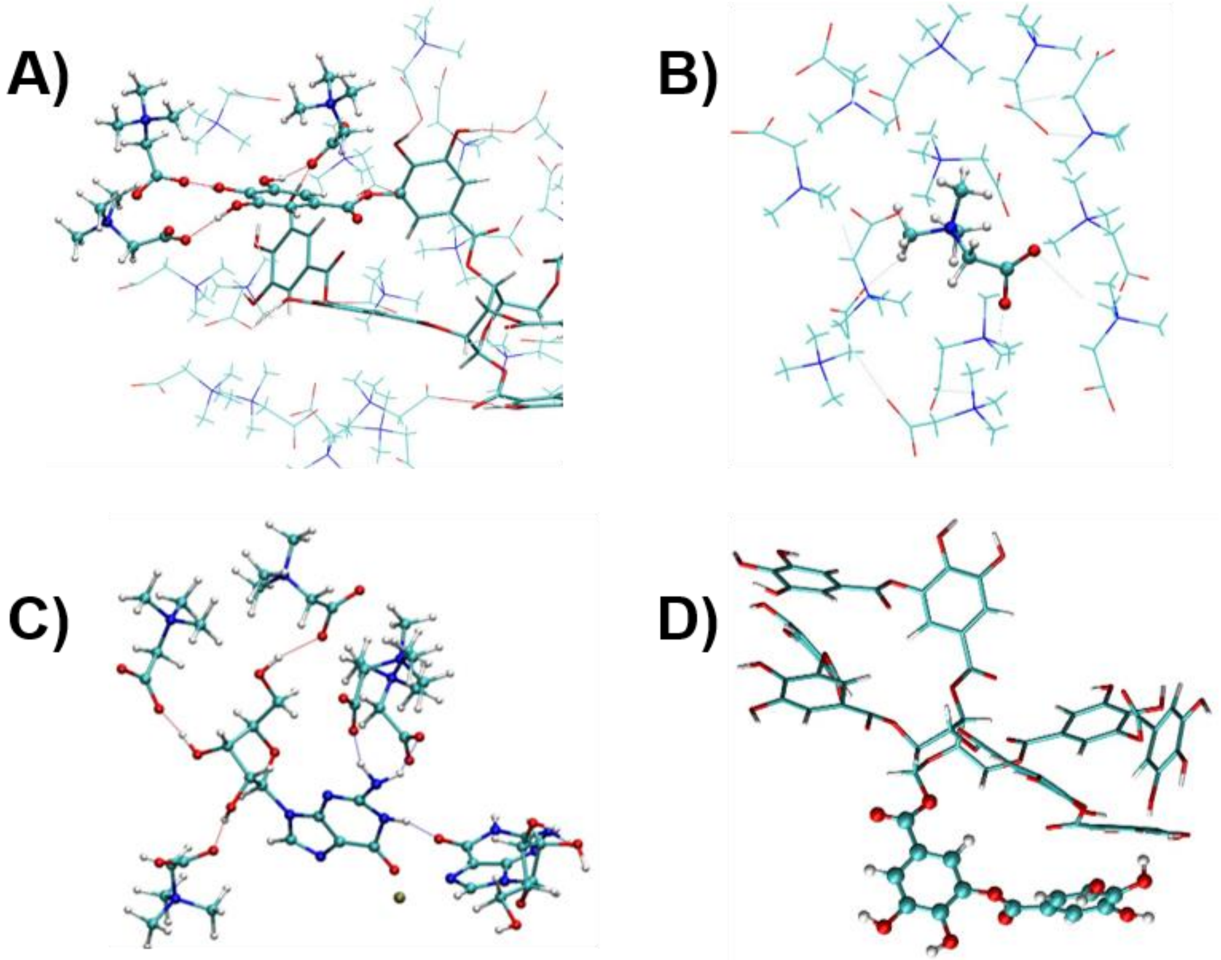

Figure S3. Representative snapshot showing Bet molecules interacting with TA, G, and other Bet molecules. (A) Hydrogen bonding between the -COO group of Bet and one of the gallic groups of a TA molecule. (B) A Bet molecule is surrounded by other Bet molecules. (c) Hydrogen bonding between a G molecule and surrounding Bet molecules. (d) One of the five phenolic moieties of a TA molecule is presented in ball-and-stick representation.

The intramolecular interaction is also strong, resembling a strong hydrogen bond (quasi-covalent) interaction. A different situation is observed in (ii). Here, the G molecules interact strongly with TA molecules and remain attached to them, precluding the G diffusion necessary for forming the tetramer structure. As shown in Figure S4, if the G molecule becomes trapped among the TA molecules, it will hardly diffuse from that site. Nevertheless, we cannot rule out the possibility of tetramer formation. Since this event requires a longer simulation time than the one conducted in our study, we could not observe the G4 formation.

4- It is not clear from our simulations the role of individual G molecules or the tetramer arrangement in the particular properties experimentally observed, but this deserves a more extensive study.

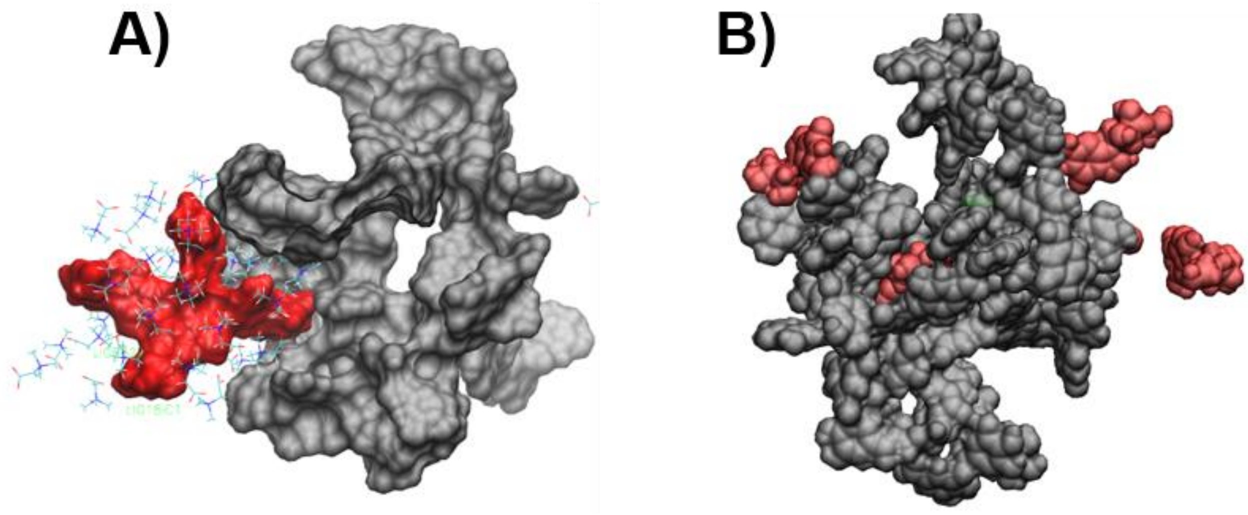

Figure S4. (A) The G4 is presented in red color and surrounded by a shell of Bet (stick representation, 3 Å to the Bet surface) and TA molecules (surface representation in gray color). (B) Here, the four guanosine molecules are separated. The rest of the molecules were removed to highlight the details.

We performed a molecular dynamics simulation with one TA molecule and 690 Bet molecules to rationalize the TA molecule interaction with Bet molecules without G

molecules. After 10 ns of simulation, we extracted the TA molecule and a shell of surrounding Bet molecules closer than 3 Å, see Figure S5. Then, we performed a structural minimization of the TA molecule, keeping the surrounding Bet molecules fixed. We found that the hydrogen bond between TA and Bet molecules is well reproduced by the force field used in this work. The computational setup can be found in the computational methods. Figure S5 shows the TA molecule and the shell of the Bet molecules.

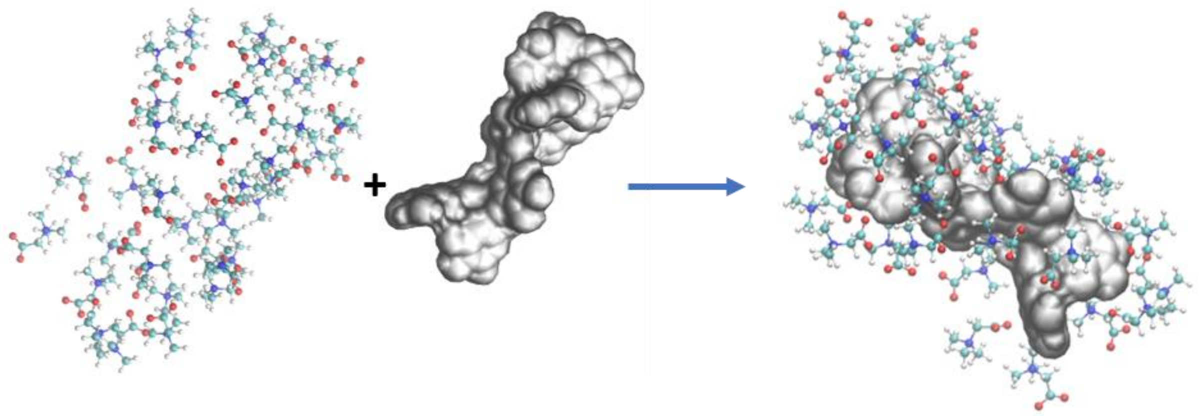

Figure S5. Snapshot taken from the molecular dynamics simulation showing the TA molecule (surface representation in color gray) surrounded by a shell of Bet molecules within 3 Å. The rest of the molecules were removed to highlight the details. The left panel shows the TA molecule separated from the shell of the Bet molecules.

We found that the Bet molecules form a shell around the TA molecules with a strong hydrogen bond interaction Bet-TA. The Bet molecules act as hydrogen bond acceptors for the -OH groups of the TA molecule. Besides, the electrostatic interaction between Bet molecules is weak due to the shielding effect produced by its methyl groups but sufficient to induce a nearly uniform film around the TA molecules. Figure S6 shows the arrangement of seven TA molecules surrounded by Bet molecules, and detail shows the

Bet molecules placed between TA molecules. These results are similar to other reported works, such as Abranches *et. al.*[5]

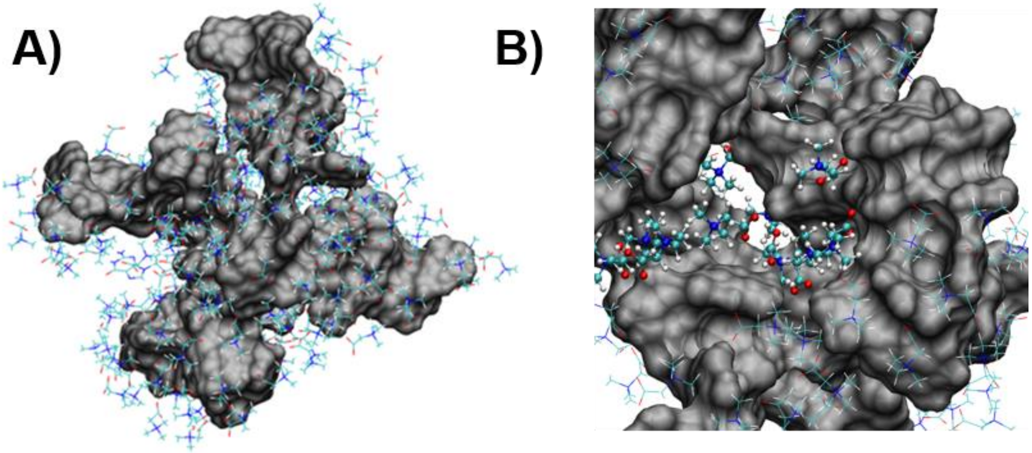

Figure S6. (A) The TA molecules (surface representation in color gray) are surrounded by Bet molecules. (B) detailed image showing the Bet molecules present in between the TA molecules.

TA – ChCl - G

1- Similar to the case with Bet, the nine carbonyl groups of TA molecules are buried and exhibit weak interactions with the chloride anion and the choline cation. Consequently, their IR frequencies are minimally affected by the presence of ChCl. The electronegative chloride anion shields the TA molecule from the choline cation, resulting in a surrounding shell of molecules that is less dense than what we observe with Bet molecules. These anions are located close to the twenty-five -OH moieties of one TA molecule (see Figure S7). Additionally, the diffusion of these chloride ions is hindered by their interaction with the hydroxyl groups of the TA molecules. The number of Cl anions surrounding a TA molecule is 18 ± 2.

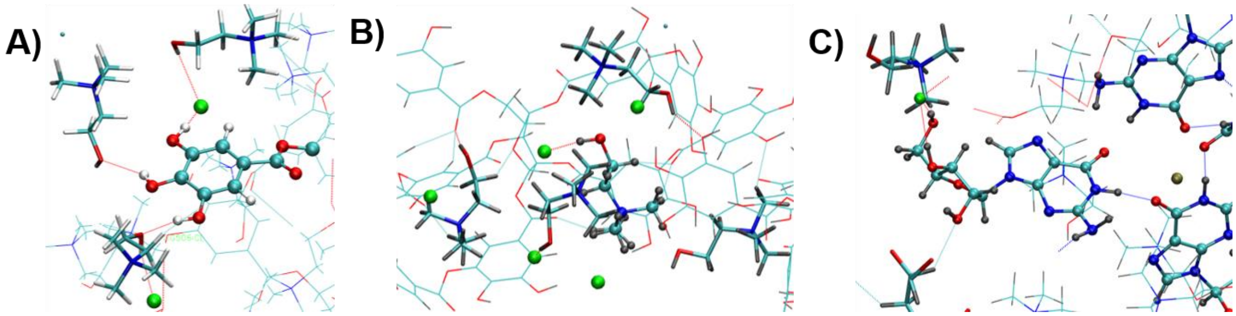

Figure S7. (A) Snapshot from the molecular dynamics simulation showing one of the gallic moiety of the TA molecule interacting with a Cl ion (green) and the choline cation. (B) A ChCl molecule surrounded by other ChCl molecules. C) One of the G molecules of the tetramer arrangement is shown surrounded by ChCl molecules. The K atom is shown in dark yellow.

The G molecules interact with TA molecules similarly to when Bet molecules are present. Figure S8 shows the tetramer arrangement, which remains structurally stable during the simulation time. We did not initiate the molecular dynamics simulation with the G molecules far away from each other, as we did for Bet molecules, but based on the results observed with Bet molecules, we believe the tetramer arrangement could occur under such initial conditions.

The role of Cl^- ions is twofold: they disrupt the intramolecular TA-TA, TA-Ch^+ cation, TA-G, and G-G interactions, thereby producing a shielding effect and reducing the density of the mixture, see Figure S8 and S9. This shielding effect could presumably be responsible for the elastic response of the TA-G-ChCl system and the dissociation of the G4 structure. Figure S10 displays the G4 molecules surrounded by ChCl molecules. During the simulation, the G4 adopts a fluctuating arrangement of hydrogen bonds between molecules or situations where one or two G molecules move apart and lose one or two hydrogen bonds, see Figure S9 and Figure S10.

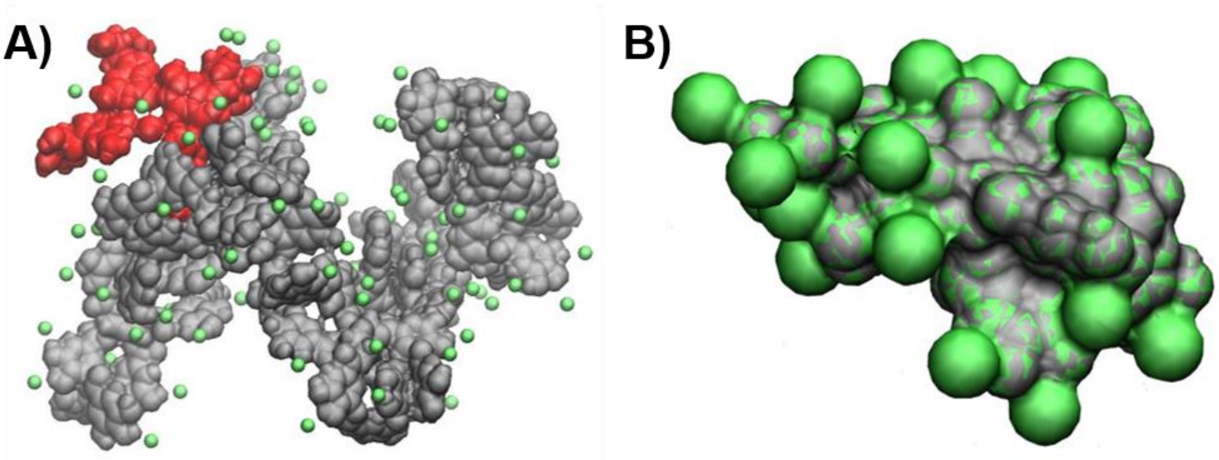

Figure S8. (A) shows the G4 (color red) interacting with TA molecules (color gray). The Cl anions are shown in green, and the remaining molecules were removed to help visualization. (B) One TA molecule surrounded by a shell of Cl ions placed closer than 4 Å (green surface representation).

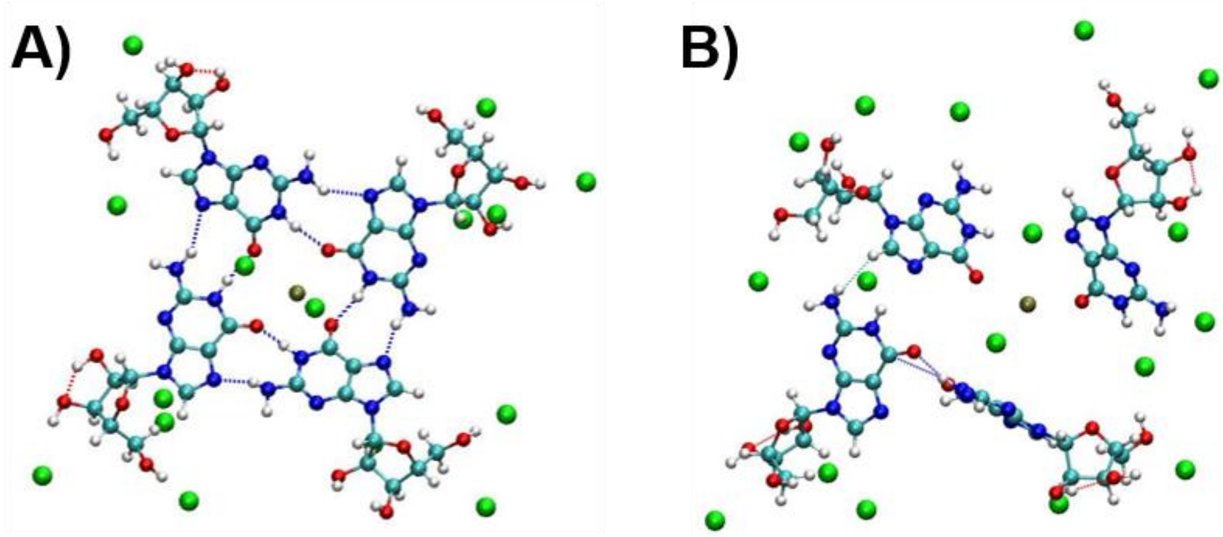

Figure S9. (A) shows the G4 structure, including the K ion, and surrounded by Cl ions taken from the MD simulation. (B) Idem (A) but showing the four G molecules with the hydrogen bond interactions disrupted by the Cl ions.

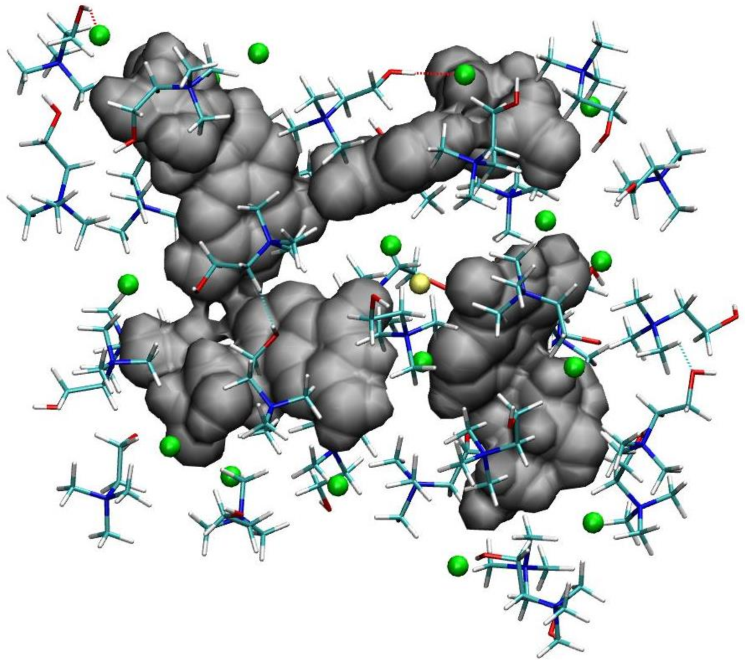

Figure S10. G4 is surrounded by ChCl molecules closer than 4 Å. The Cl ions are shown in green, and the K ion is represented in yellow.

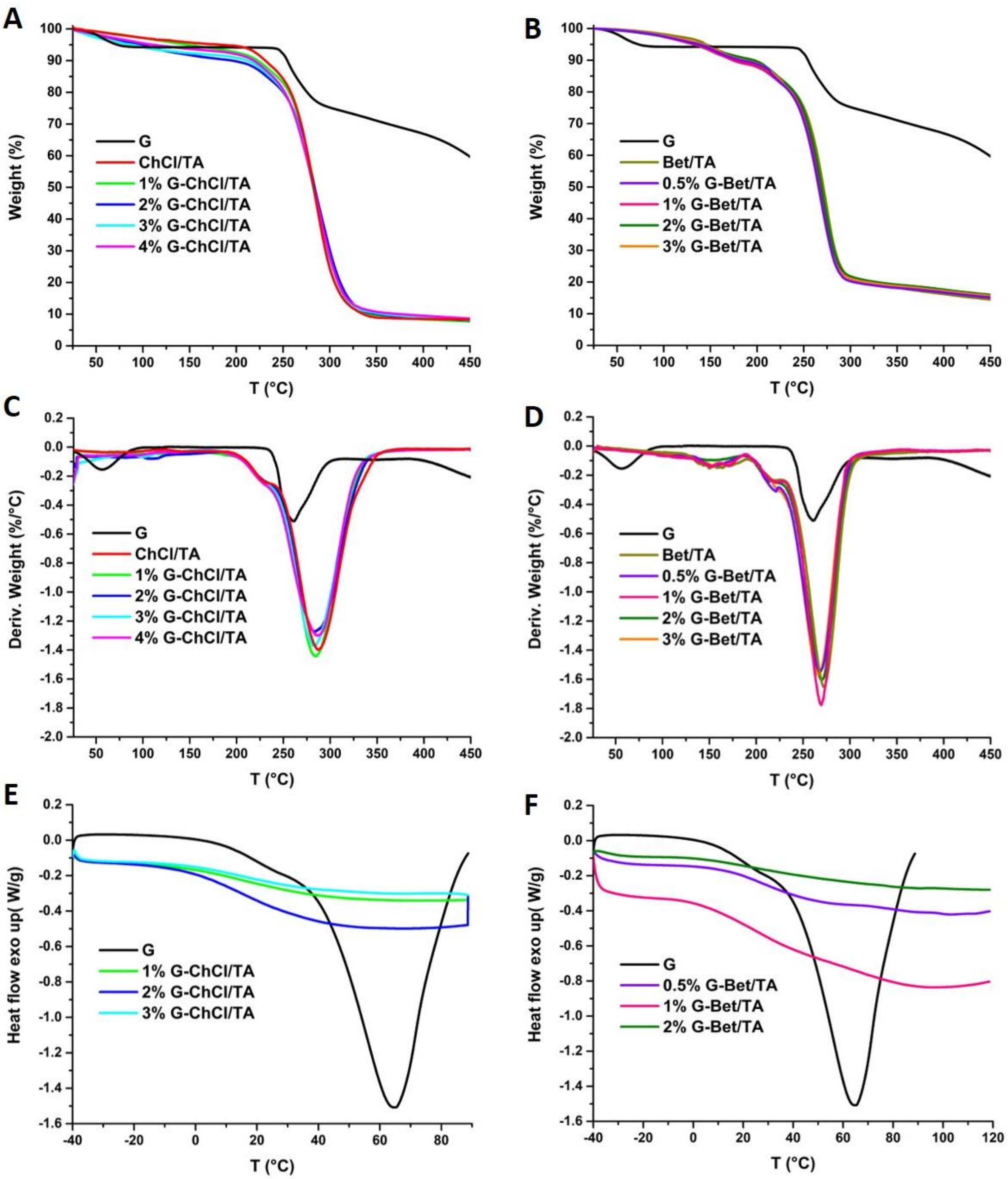

Figure S11. TGA curves (A and B), calculated derivative weight %/°C (C and D), and DSC scans of the heating cycle of TA-based LTTMs/G samples (E and F).

Table S2. Thermal properties of the G powder, LTTMS, and solvent-free supramolecular adhesive systems.

Sample	$T_{5\%}$	$T_{50\%}$	$T_{max\%}$	T_g
G	72.8	-	256.2	-
ChCl/TA	182.4	285.3	287.3	-
1% G-ChCl/TA	147.1	283.8	284.1	16.6
2% G-ChCl/TA	96.9	283.8	282.5	15.3
3% G-ChCl/TA	89.5	274.1	281.1	16.8
4% G-ChCl/TA	110.5	281.9	288.3	34.2
Bet/TA	145.3	270.1	273.8	20.8
0.5% G-Bet/TA	137.8	266.1	268.1	21.6
1% G-Bet/TA	132.2	267.5	269.4	21.2
2% G-Bet/TA	136.6	268.9	269.9	22.9
3% G-Bet/TA	141.6	265.7	266.8	23.2

$T_{5\%}$: degradation temperature at 5%
$T_{50\%}$: degradation temperature at 50%
$T_{max\%}$: maximum degradation temperature
T_g: glass transition temperature

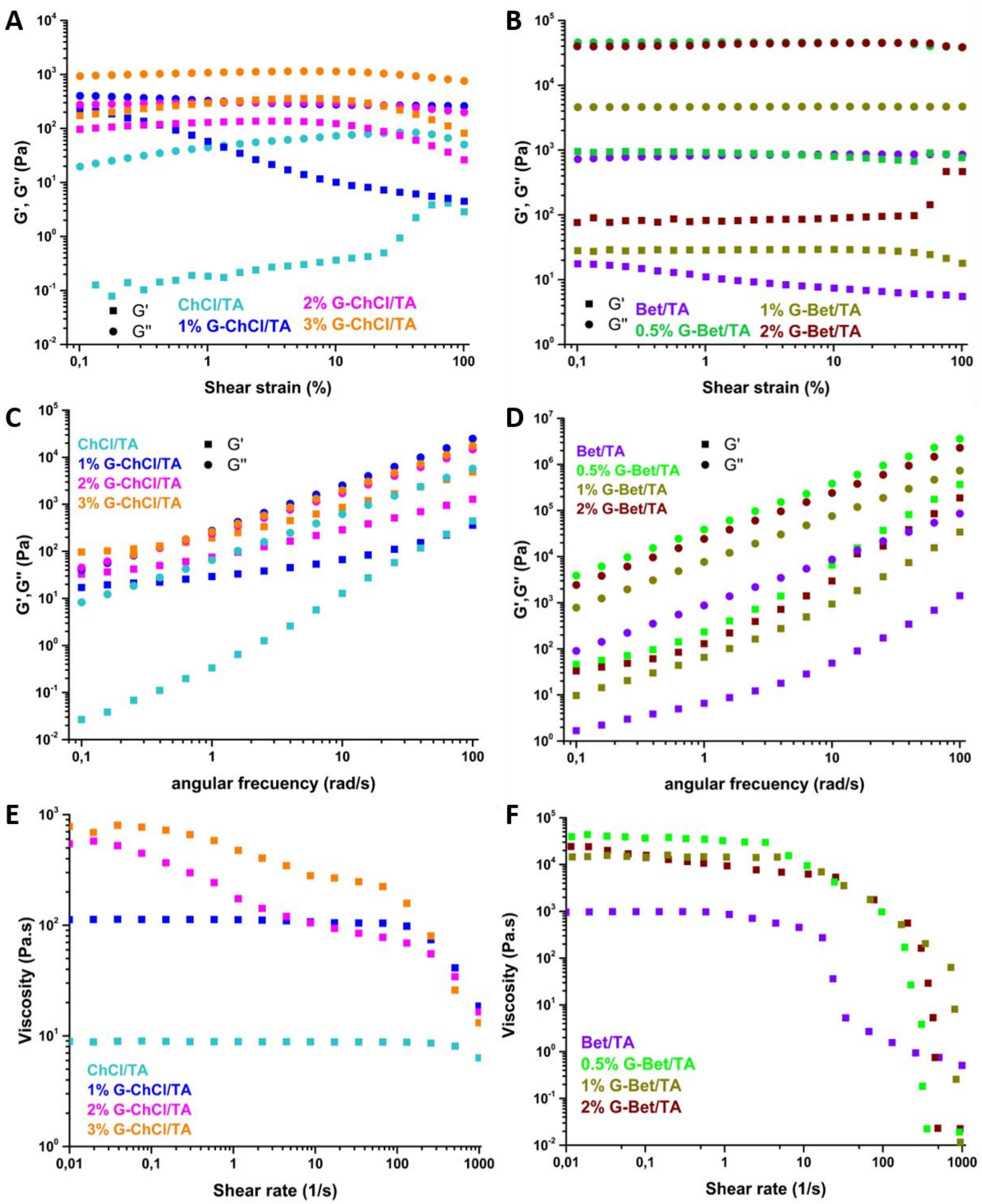

Figure S12. Strain sweep from 0.1 to 100% at a frequency of 1.0 rad s^{-1} for the supramolecular adhesives fabricated with ChCl/TA (A) and Bet/TA (B) LTTMs. Frequency sweep analysis from 0.1 to 100 rad/s at a constant strain of 0.1% for the supramolecular adhesives fabricated with ChCl/TA (C) and Bet/TA (D) LTTMs. Viscosity as a function of the shear rate for the supramolecular adhesives fabricated with ChCl/TA (E) and Bet/TA (F) LTTMS.

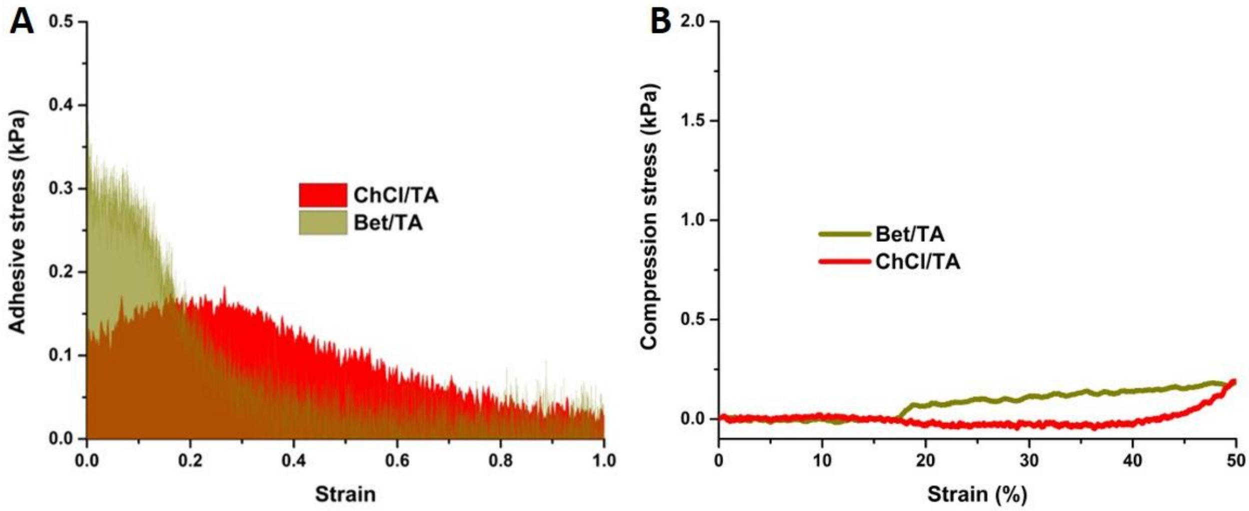

Figure S13. A) Adhesive stress vs. strain curves for the ChCl/TA and Bet/TA LTTMs. B) Stress vs. strain compression curves for the ChCl/TA and Bet/TA LTTMs.

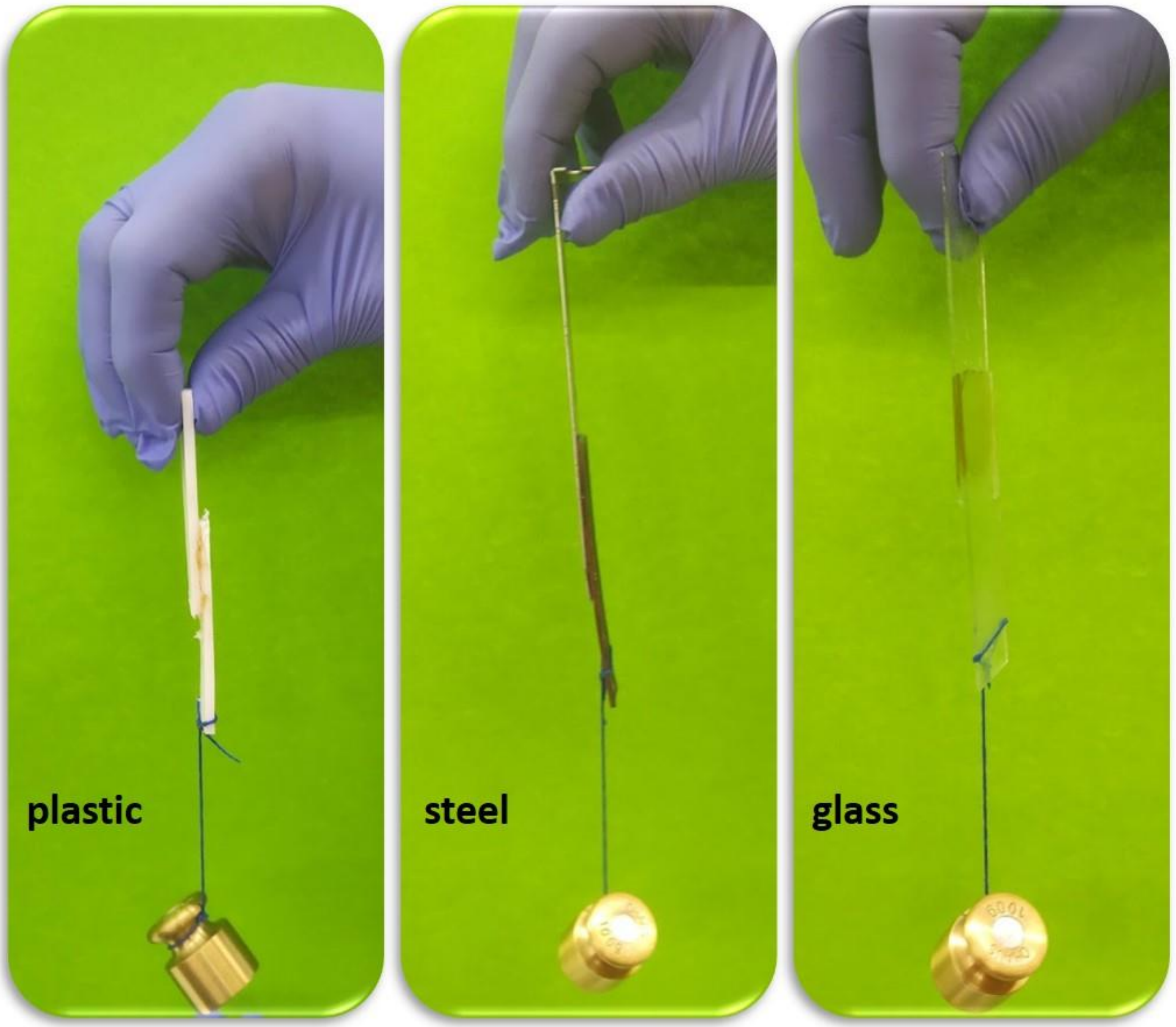

Figure S14. Adhesion behavior of the 3% G-ChCl/TA adhesive for attaching two identical substrates.

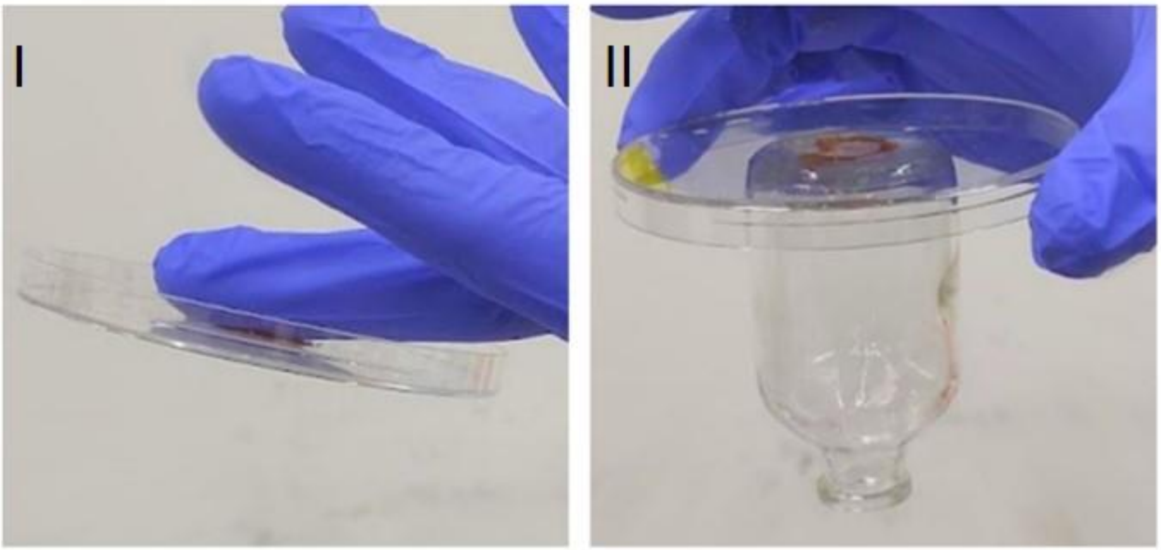

Figure S15. Adhesion behavior of the 2% G-Bet/TA adhesive for attaching nitrile to plastic (I) or plastic to glass (II).

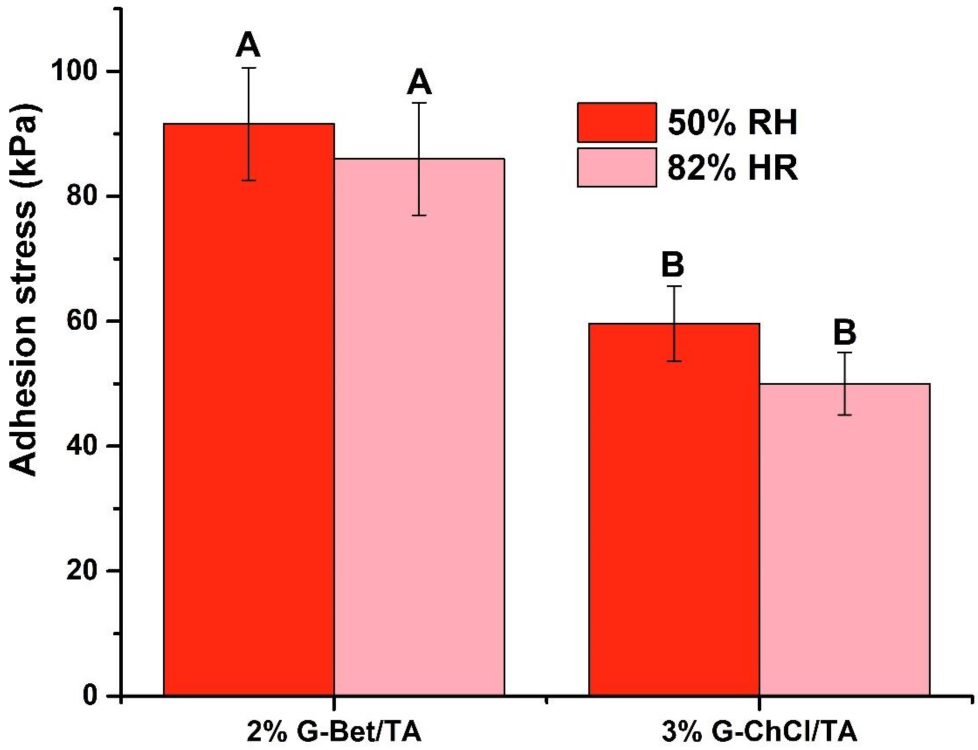

Figure S16. Adhesion stress of the 2% G-Bet/TA and 3% ChCl-Bet/TA adhesives on pigskin substrates at 50% and 82% RH.

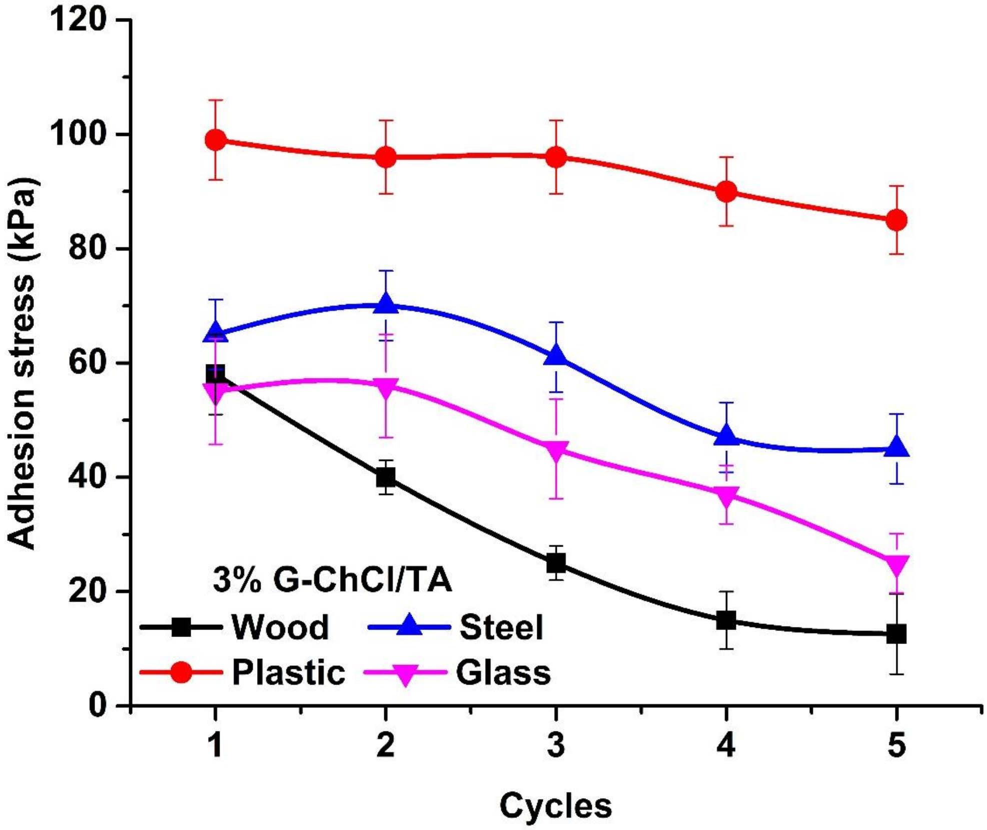

Figure S17. Cyclic adhesion test of 3% G-ChCl/TA sample on different substrates.

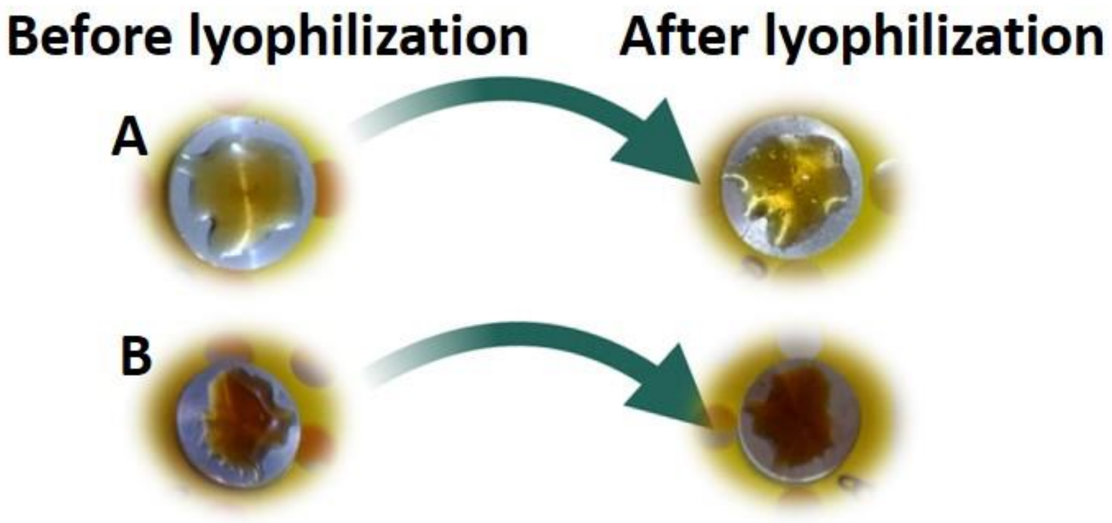

Figure S18. Photography of 3% G-ChCl/TA (A) and 2% G-Bet/TA (B) after and before 24 h of lyophilization**.**

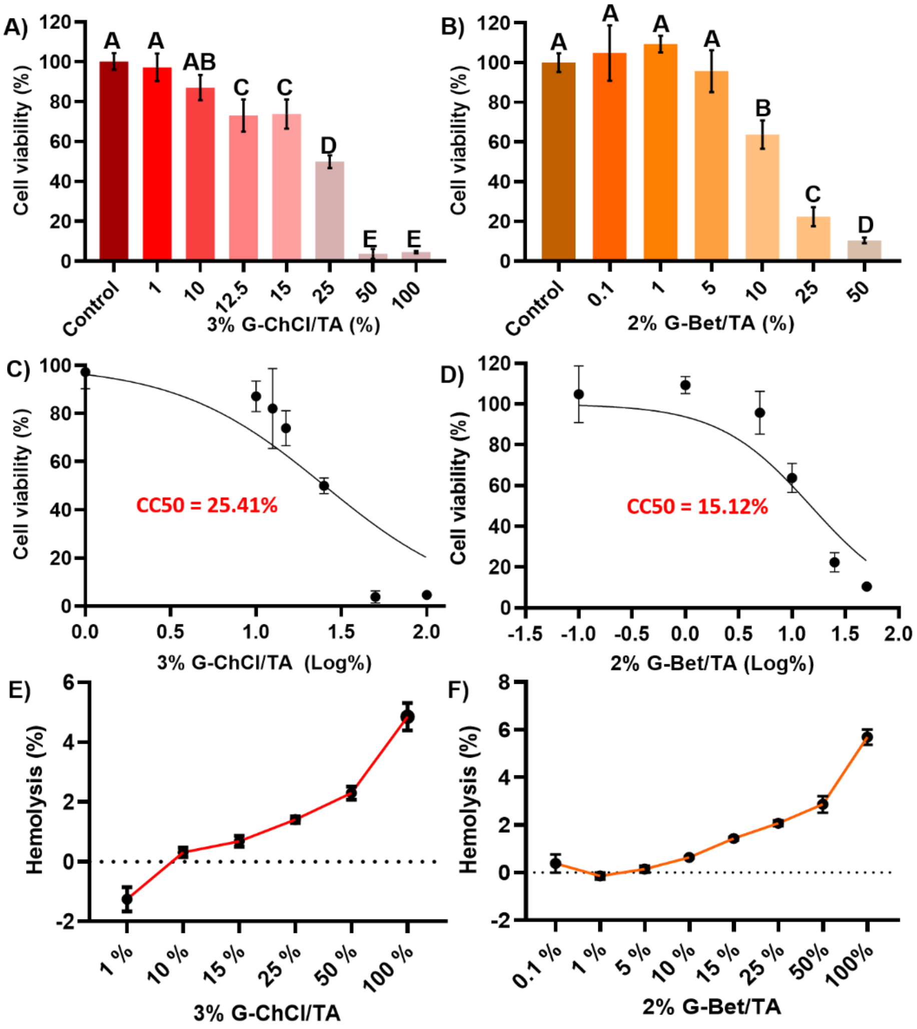

Figure 19. Cytotoxicity testing of S-F_SAS extracts on MRC-5 human fibroblast cells by flow cytometry. MRC-5 human fibroblast cells were exposed to increasing concentrations of 3% G-ChCl/TA and 2% G-Bet/TA extracts during 24 h and cell viability was analyzed using live–dead cell staining. Cells exposed only to the culture medium were used as the control. Results are given as the percentage of live cells exposed to 3% G-ChCl/TA (A) or 2% G-Bet/TA (B) extracts compared to the control. The viability of the cells exposed only to the culture medium was set at 100% (Control). Results given as the log_{10} of the percentage of live cells exposed to 3% G-ChCl/TA (C) or 2% G-Bet/TA (D) extracts compared to the control. Percentage of hemolysis of 10% human erythrocyte suspension (RBC) after incubation with different concentrations of 3% G-ChCl/TA (E) or 2% G-Bet/TA (F) extracts for 30 min at 37 °C.Hemolyzed RBC with 10% Triton X-100 was used as the positive control (100% lysis) and RBC exposed to PBS alone was used as the negative control. Data are expressed as mean ± SD and represent three experiments with similar results performed in triplicate. Two bar values with the same letter are not significantly different ($p \geq 0.05$) according to Tukey's test (n=3). Median Cytotoxic Concentration (CC50) is the concentration of S-F_SAS extract that causes cytotoxicity in 50% of the cells.

References

(1) Chai, J.-D.; Head-Gordon, M. Systematic Optimization of Long-Range Corrected Hybrid Density Functionals. *J. Chem. Phys.* **2008**, *128*. https://doi.org/10.1063/1.2834918.

(2) Phillips, J. C.; Braun, R.; Wang, W.; Gumbart, J.; Tajkhorshid, E.; Villa, E.; Chipot, C.; Skeel, R. D.; Kalé, L.; Schulten, K. Scalable Molecular Dynamics with NAMD. *J. Comput. Chem.* **2005**, *26*, 1781–1802. https://doi.org/10.1002/jcc.20289.

(3) Petry, R.; Focassio, B.; Schleder, G. R.; Martinez, D. S. T.; Fazzio, A. Conformational Analysis of Tannic Acid: Environment Effects in Electronic and Reactivity Properties. *J. Chem. Phys.* **2021**, *154*. https://doi.org/10.1063/5.0045968.

(4) Sæbø, I.; Bjørås, M.; Franzyk, H.; Helgesen, E.; Booth, J. Optimization of the Hemolysis Assay for the Assessment of Cytotoxicity. *Int. J. Mol. Sci.* **2023**, *24*, 2914. https://doi.org/10.3390/ijms24032914.

(5) Abranches, D. O.; Silva, L. P.; Martins, M. A. R.; Pinho, S. P.; Coutinho, J. A. P. Understanding the Formation of Deep Eutectic Solvents: Betaine as a Universal Hydrogen Bond Acceptor. *ChemSusChem* **2020**, *13*, 4916–4921. https://doi.org/10.1002/cssc.202001331.
